# Foraminifera assemblages from Fantangisña serpentinite mud seamount in the NW Pacific Ocean during the Pleistocene (IODP Expedition 366)

**DOI:** 10.1002/jqs.3532

**Published:** 2023-05-30

**Authors:** Arianna V. Del Gaudio, Werner E. Piller, Gerald Auer, Walter Kurz

**Affiliations:** ^1^ Institute for Earth Sciences, NAWI Graz Geocenter University of Graz Heinrichstrasse 26 Graz 8010 Austria

**Keywords:** benthic foraminifera, North Equatorial Current, planktonic foraminifera, Pleistocene, serpentinite mud volcanism

## Abstract

The Mariana forearc system, in the northwestern Pacific, is known as the only convergent margin setting with currently active serpentine mud volcanism. The Fantangisña serpentinite mud volcano lies 62 km west of the Mariana trench, within the influence of the North Equatorial Current (NEC). Cores recovered by International Ocean Discovery Program (IODP) Expedition 366 contain pelagic sediments overlying layered serpentinite mud deposits. At the bottom of the sequence, nannofossil‐rich forearc deposits were recovered from under the seamount edifice. In this study, we investigated 47 samples from Site U1498A on the southern flank of the seamount for benthic and planktonic foraminifera assemblages. Statistical analyses on planktonic assemblages differentiated two sample groups related to the ratio between thermocline/mixed layer taxa, which indicate fluctuations in the depth of the thermocline (DOT) during the Pleistocene. Variations in the DOT reflect changes in the intensity of the NEC associated with El Niño/La Niña conditions. Mudflows do not influence the ecology of planktonic foraminifera but possibly enhance their preservation against dissolution, which was instead detected in the pelagic deposit as suggested by common *Globigerinoides conglobatus*. Benthic foraminifers were rare in serpentinite mud deposits as they are severely affected by mudflows. Conversely, they showed high diversity pre‐/post‐mud‐volcanism, and indicate oligotrophic bottom‐water conditions.

**Abbreviations**
DOT: depth of the thermocline
EMPT: early‐middle pleistocene transition
ENSO: El Niño‐southern oscillation
IBM: Izu‐Bonin‐Mariana
IODP: International ocean discovery program
KC: Kuroshio current
m: meter
Ma: million year
mbsf: meters below the sea floor
mbsl: meters below the sea level
MIS: marine isotope stage
m/Myr: meters per million years
NEC: north equatorial current
nMDS: non‐metric multidimensional scaling
NPSG: north pacific subtropical gyre
NW: Northwestern
ODP: ocean drilling program
PAST: PAleontological STatistics
PCA: principal component analysis
PC: principal component
SEM: scanning electron microscope
SIMPER: similarity percentage
s.l: sensu lato
s.s.: sensu strictu
SST: sea surface temperature
Sv: Sverdrup
UPGMA: unweighted paired group
WC: Walker circulation

## Introduction

**Figure 1:**
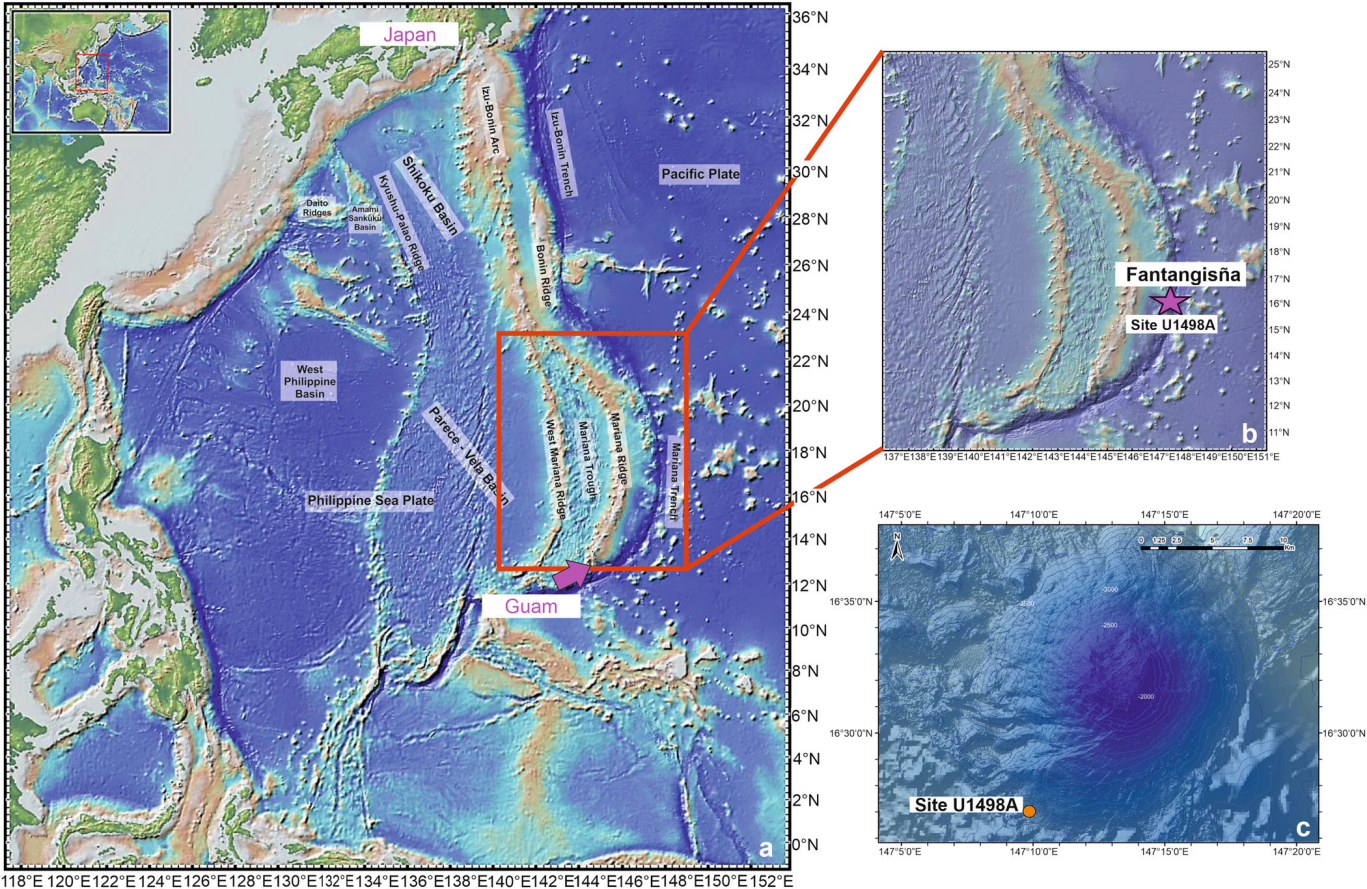
(a) Bathymetric map of the Izu–Bonin–Mariana system. (b) Close‐up illustrating the location of Site U1498A. (c) Elevation map of Fantangisña seamount. Modified after Del Gaudio et al., 2022. [Color figure can be viewed at wileyonlinelibrary.com]

The Mariana forearc, in the northwestern Pacific Ocean, constitutes the southern sector of the Izu–Bonin–Mariana (IBM) trench–arc system (12°N to 35°N), an intra‐oceanic convergent margin formed by the subduction of the Jurassic–Cretaceous west Pacific Plate underneath the Eastern Eurasian margin and the Philippine Plate (Uyeda and Kanamori, 1979; Uyeda, 1982; Fryer, 1996; Stern et al., 2003; Kurz et al., 2019; Reagan et al., 2019; Deng et al., 2021). Subduction started in the Eocene ~50–52 million years ago (Ma) (Uyeda and Ben‐Avraham, 1972; Karig, 1975; Fryer et al., 1990; Cosca et al., 1998; Menapace et al., 2019; Reagan et al., 2019). The IBM stretches for approximately 2800 km (Figure 1a) from Japan to the Mariana islands, situated south of Guam (Stern et al., 2003).

Sixteen seamounts representing serpentinite mud volcanoes were discovered on the Mariana forearc at different distances (30–90 km) from the trench (Wheat et al., 2010). The seamounts are 30–50 km wide and 2–3 km high, covering an area of 100 km^2^ (Hussong and Fryer, 1985; Fryer et al., 1985; Fryer, 1992). The serpentinite mud edifices in the Mariana region result from the only known active serpentinisation of the upper plate lithospheric mantle in a convergent margin setting (Fryer, 2012; Fryer et al., 2000). Slab‐derived fluids (Fryer et al., 2020), formed from dehydration reactions occurring in the subducted Pacific Plate (Peacock, 1990; Mottl, 1992; Schmidt and Poli, 1998; Hyndman and Peacock, 2003; Rupke et al., 2004; Curtis et al., 2013; Fryer et al., 2020), serpentinise the Mariana forearc mantle, generating unconsolidated serpentinite mud (Fryer, 2012). The formation of the serpentinite mud volcanoes is therefore related to episodic extrusions of serpentinite muds to the seafloor which, in turn, is linked to the upwelling of fluids from the slab (Fryer, 2012).

**Figure 2:**
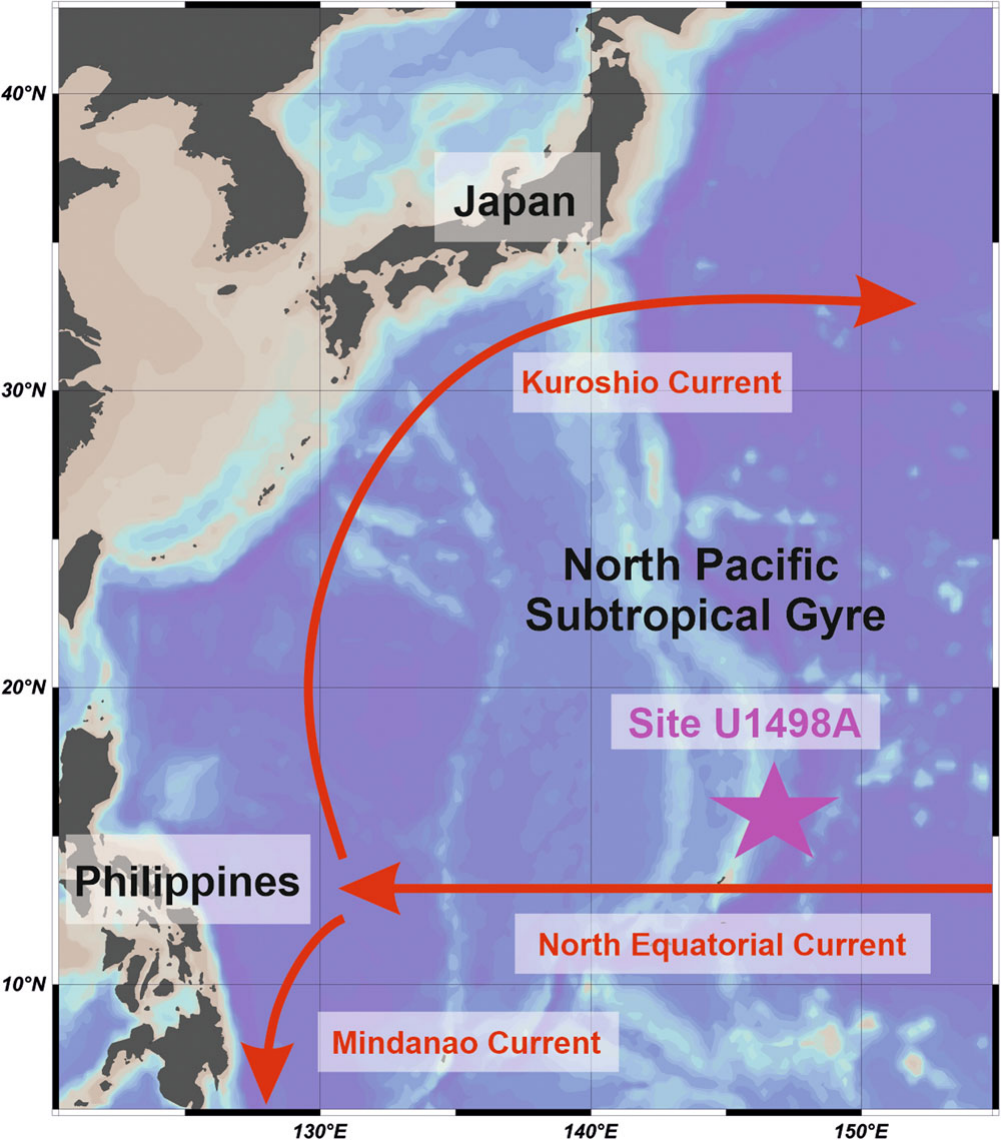
Map showing the major oceanic currents mentioned in the text. Map created using Ocean Data View (Schlitzer, 2021). [Color figure can be viewed at wileyonlinelibrary.com]

Mud volcanism in the region is strictly connected to fault systems on the Mariana forearc (Menapace et al., 2019). Those faults represent the preferable pathway for the serpentinite mud to migrate upwards and successively extrude to the seafloor, forming the large serpentinite seamounts (Fryer, 1992). Three serpentinite mud volcanoes (Yinazao, Fantangisña and Asùt Tesoru) were cored on the Mariana forearc during International Ocean Discovery Program (IODP) Expedition 366 on a south–north transect at 55–72 km from the trench (Fryer, 2018a). This study focuses on material from Site U1498A, cored on the Fantangisña serpentinite mud volcano (Figures 1b and 1c). The seamount is situated at low latitudes in the northwestern Pacific Ocean, under the influence of the North Equatorial Current (NEC) (Figure 2), a warm and oligotrophic equatorial surface water mass (Cabrera et al., 2015) that flows westward in the tropical Pacific Ocean, and is driven by the trade winds (Kubota et al., 2021).

El Niño–Southern Oscillation (ENSO) events (Kim et al., 2004; Lukas, 1996) affect wind strength and the velocity of the NEC. In turn, changes in NEC intensity are strongly linked to the depth of the thermocline (DOT) in the western Pacific (Liu and Zhou, 2020).

This study presents the first evidence of the distribution and ecology of planktonic and benthic foraminifera assemblages and their preservation in the highly dynamic depositional setting of an active serpentinite mud volcano. Our goals were to understand: (1) whether the serpentinite mud volcanism affects the ecological distribution and preservation of planktonic foraminifera; (2) whether the changes in planktonic assemblages during the Early–Late Pleistocene are associated with fluctuations of the NEC related to ENSO climate phases (El Niño/La Niña); (3) the response of benthic foraminifera to the serpentinite mud activity by recording variations in their total abundance; and (4) bottom‐water conditions before and after the serpentinite mudflow activity, by analysing the benthic assemblages.

### Geological setting

Fantangisña represents a large serpentinite mud volcano (20 km diameter, 1600 m height) (Oakley, 2007; Menapace et al., 2019) located at 16°32.25′N, 147°13.25′E (Fryer et al., 2018b). It formed on an uplifted forearc block with a northwest inclination (Fryer et al., 2018b) and is situated on the Mariana forearc, 62 km west of the Mariana Trench (Wheat et al., 2020; Fryer et al., 2018b). At the top of the edifice, normal faulting induced the formation of a 5 km long, 3.5 km wide, U‐shaped depression (Oakley, 2007).

Menapace et al. (2019) attempted to assess the timing of the onset of Fantangisña serpentinite mud volcanic activity using combined planktonic foraminiferal and calcareous nannofossil biostratigraphy on samples collected during IODP Expedition 366. Their proposed age for the emplacement of the seamount was estimated at 10.77 Ma (Menapace et al., 2019). A higher‐resolution biostratigraphic study from Del Gaudio et al. (2022) constrained the latest phase of Fantangisña to between 6.10 Ma (Late Miocene) and 4.20 Ma (Early Pliocene), 6 Ma younger than the estimated age suggested by Menapace et al. (2019). Del Gaudio et al. (2022) did not consider the interval mentioned above as indicative of the onset of the volcanic activity for Fantangisña but rather as its most recent phase of serpentinite mud production. This difference is because the analysed cores were drilled near the toe of the seamount, far from the conduit, where older serpentinite mudflows may be present. Structural geological studies (Frery et al., 2021) indicate that the mud volcanoes were episodically built from the start of the subduction to the present day as a function of the forearc tectonic activity. Interestingly, the latest stage of Fantangisña seamount activity was found to be concurrent with the onset of the rifting in the Mariana Trough (Del Gaudio et al., 2022) occurring at 7–6 Ma (Clift and Lee, 1998; Sato et al., 2015; Anderson et al., 2017).

Two sites, U1498 and U1497, were drilled on the flank and at the top of the seamount, respectively (Fryer et al., 2018a; Figure 1c). For Site U1498, Holes A and B were drilled (Fryer et al., 2018b), and 181.6 m (11.34% recovery) and 260 m (31.85% recovery) were cored, respectively. We focused our planktonic and benthic foraminifera analyses on Site U1498 Hole A as it recovered the most complete stratigraphic succession among all sites. This site also yielded the lowest number of barren samples.

### Oceanographic setting

Site U1498A is located at the southern margin of the North Pacific Subtropical Gyre (NPSG; Figure 2) in the northwestern sector of the Pacific Ocean. The NPSG plays a key role in the heat transport within the ocean and is responsible for the surface and intermediate water circulation in the northern part of the basin (Ujiié et al., 2016). The NPSG is a clockwise flow bounded by four major boundary currents. Among them, the Kuroshio Current (KC) and the NEC (Figure 2) represent the western and southern sides of the gyre, respectively.

The NEC is a wind‐drift current flowing east to west between 8°N and 17°N (Qiu et al., 2015), carrying warm oligotrophic waters into the western Pacific Ocean (Cabrera et al., 2015). Several studies (Toole et al., 1990; Qiu and Lukas, 1996, 2003; Kim et al., 2004; Zhai and Hu, 2012) have underlined the importance of the NEC in the supply of heat, salt and water masses to the KC through the NPSG. According to the glider section calculated by Schönau and Rudnick (2015), the mean transport of NEC is 37.6 Sverdrup (Sv; 1 Sv represents 1 million cubic metres per second) with a standard deviation value of 15.6 Sv. Numerical modelling indicates that the NEC transport and intensity are strongly linked to the ENSO circulation system on an interannual time scale (Qiu and Lukas, 1996; Wang and Hu, 2006; Liu and Zhou, 2020). In the modern western Pacific Ocean, the NEC volume transport and its intensity are reduced during the El Niño intervals, while the NEC intensifies during the La Niña phases (An et al., 2018). These changes are related to wind anomalies in the western‐central tropical North Pacific Ocean (Qiu and Joyce, 1992; Qiu and Chen, 2010; Zhai and Hu, 2012). During El Niño/La Niña, the regular pattern of the atmospheric circulation in the Pacific Ocean (known as ‘Walker circulation’ (WC)) is modified with trade winds weakening/strengthening over the western Pacific (Kaboth‐Bahr and Mudelsee, 2022). Recent studies indicated that the shift from pre‐modern to modern‐like WC likely happened during the Middle Pleistocene (Kaboth‐Bahr and Mudelsee, 2022).

Near the Philippine coast (western Pacific Ocean), the NEC separates into the KC to the north and the Mindanao Current turning to the south (Qiu et al., 2015; Lam et al., 2021; Figure 2). Subsequently, the KC moves along the coast of Japan. At approximately 36°N, 141°E, the KC diverges from Japan, becoming the Kuroshio Current Extension (Lam et al., 2021). Our target site, U1498A, is situated within the latitudinal band of the NEC (Figure 2).

## Materials and methods

### Study site

**Figure 3:**
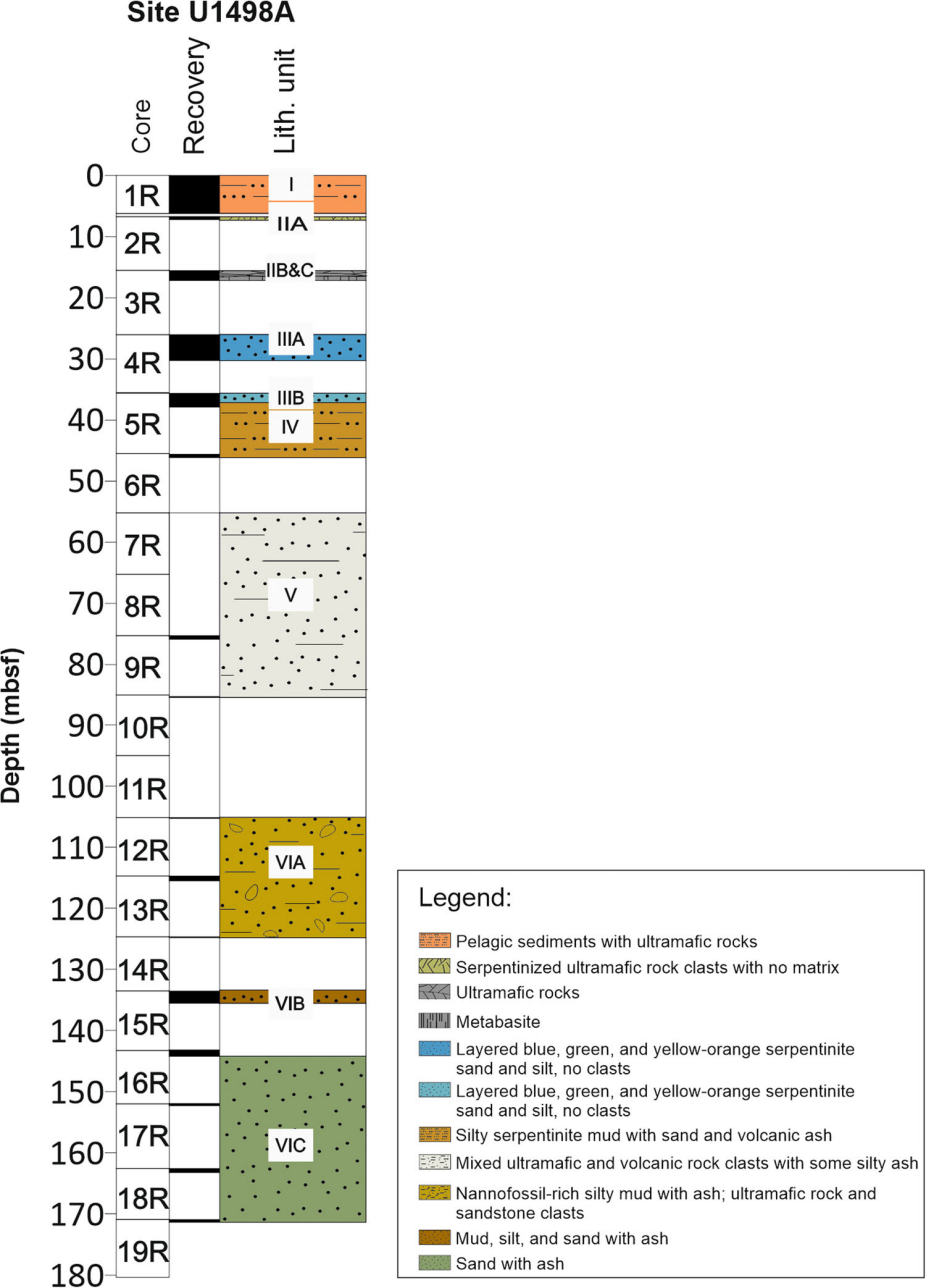
Lithology at Site U1498A (modified from Fryer et al., 2018b). The white bars represent intervals of core non‐recovery. [Color figure can be viewed at wileyonlinelibrary.com]

Site U1498 Hole A (3496.21 m water depth) was cored on the most stable southwestern flank of Fantangisña seamount (Fryer et al., 2018a; see Figure 1c). The lithological and biostratigraphic data are from Fryer et al. (2018b) and Del Gaudio et al. (2022). The stratigraphic sequence at Site U1498A shows pronounced variability in lithology (Figure 3) and is of Late Miocene (Messinian) to Quaternary age. Unit I (0–5.40 m below the seafloor (mbsf)) consists of oxidised foraminifera and nannofossil‐rich pelagic sediments with ultramafic clasts. Most of the sediments from Unit I (0.06–4.38 mbsf) were dated Middle to Late Pleistocene (0.22–0.07 Ma), the bottom part (4.38–5.25 mbsf) to 0.40–0.22 Ma (Middle Pleistocene). The average sedimentation rate for the unit is 4.83 m/Myr (Del Gaudio et al., 2022). Unit II is characterised by ultramafic rock with no matrix, making it unsuitable for micropalaeontological investigation. Layers of varicoloured serpentinite silt and sand interbedded with pelagic beds define Units III and IV (25.80–45.51 mbsf), which are Early–Middle Pleistocene in age (1.17–0.61 Ma). Sedimentation rates for Units III and IV are remarkably high (94.71 m/Myr; Del Gaudio et al., 2022). Early Pliocene to Early Pleistocene (4.20–1.17 Ma) sediments of Unit V (55.10–84.45 mbsf) represent a mix of unconsolidated silty ash, ultramafic and volcanic clasts. Unit VI (103.80–172.05 mbsf) consists of forearc silty and sandy volcanic ash deposits rich in calcareous nannofossils and represents the base of the stratigraphic sequence. Most of Unit VI (113.50–172.05 mbsf) is dated to the Late Miocene (7.10–6.10 Ma). In the upper part of Unit VI (103.80–113.50 mbsf), planktonic foraminifera and calcareous nannofossils did not enable a well‐constrained biostratigraphy, possibly containing Early Pliocene or Late Miocene fauna.

### Sample preparation

**Plate 1:**
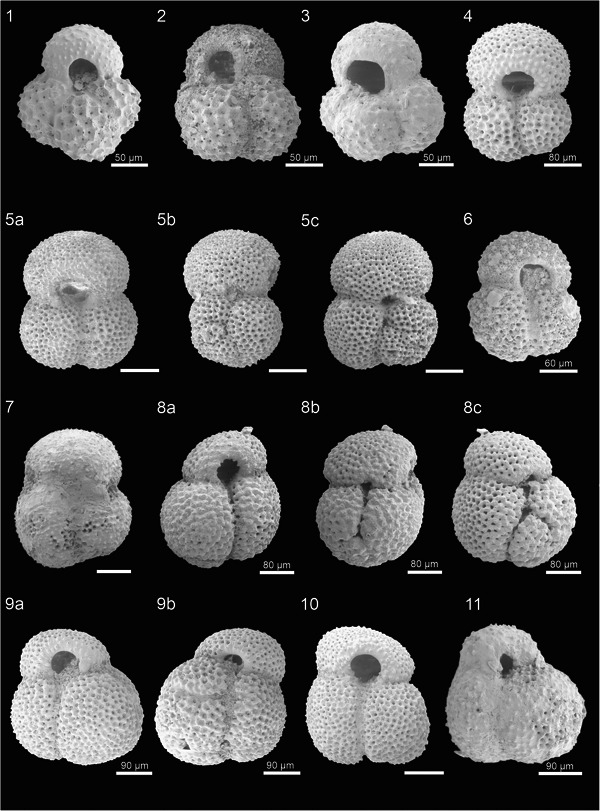
**1‐2** *Globoturborotalita rubescens* found in the pelagic unit. (1) Sample U1498A 1R‐3W, 0‐3; (2) Sample U1498A 1R‐2W, 59‐61. **3** *Globoturborotalita rubescens* taken from serpentinite mud deposits, Sample U1498A 4R‐1W, 17‐19. **4‐7** specimens of *Globigerinoides ruber* s.s. to show the variability within the *G. ruber* s.s. group. (4) Sample U1498A 1R‐3W, 61‐63; (5) Sample U1498A 1R‐2W, 59‐61 a) umbilical view; b) lateral view; c) spiral view; (6) Sample U1498A 1R‐2W, 59‐61, (7) Sample U1498A 4R‐1W, 120‐123. **8‐11** specimens of *Globigerinoides ruber* sl. to show the variability within *G. ruber* s.l. group. (8) Sample U1498A 1R‐2W, 59‐61 a) umbilical view; b) lateral view; c) spiral view; (9) Sample U1498A 1R4W, 120‐122 a) umbilical view; b) spiral view; (10) Sample U1498A 1R‐1W, 58‐60; (11) Sample U1498A 4R‐1W, 82‐84. All scale bars are 100 µm except where otherwise stated. All specimens were imaged at 5 kv and with external secondary electron (SE) detector unless otherwise noted.

**Plate 2:**
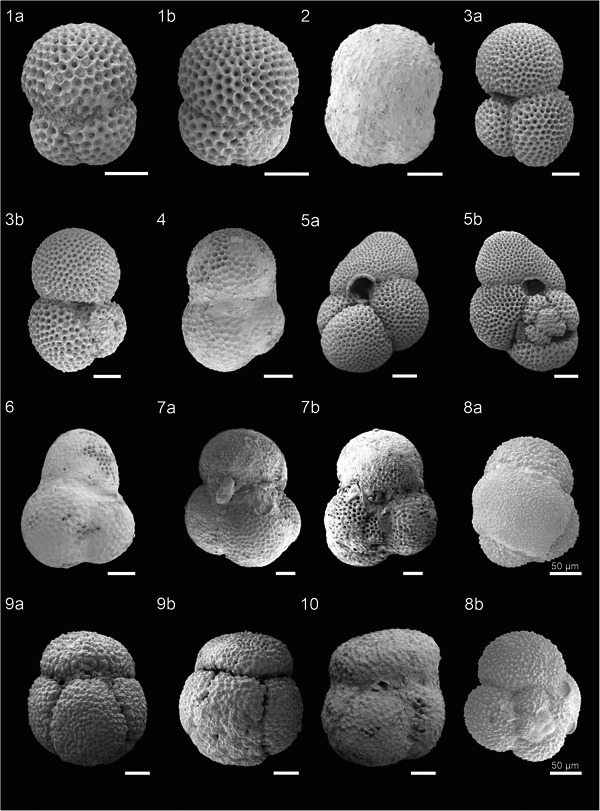
**1‐2** *Triobatus trilotus* found in the pelagic unit (1) Sample U1498A 1R‐3W, 0‐3 a) umbilical view; b) spiral view, and in the serpentinite mud deposit (2) Sample U1498A 4R‐1W, 45‐47. **3‐4** *Triobatus immaturus* in the pelagic cover (3) Sample U1498A 1R‐2W, 2‐4 a) umbilical view; b) spiral view, and in the serpentinite mud deposits (4) Sample U1498A 4R‐1W, 45‐47. **5‐6** *Triobatus sacculifer* found in the pelagic unit (5) Sample U1498A 1R‐1W, 58‐60 a) umbilical view; b) spiral view, and in the serpentinite mud deposits (6) Sample U1498A 4R‐1W, 82‐84. **7** *Trilobatus quadniobatus*, Sample U1498A 4R‐1W, 17‐19 a) umbilical view; b) spiral view. **8** *Globigerinita giutinata*, Semple U1498A 1R‐2W, 59‐61 a) umbilical view; b) spiral view. **9‐10** *Globigerinoides conglobatus* in the pelagic cover (9) Sample U1498A 1R‐1W, 6‐8 a) umbilical view; b) spiral view, and in the serpentinite mud deposit (10) Sample U1498A 5R‐3W, 31‐34. All scale bars are 100 µm except where otherwise stated. All specimens were imaged at 5 kv and with external secondary electron (SE) detector unless otherwise noted.

**Plate 3:**
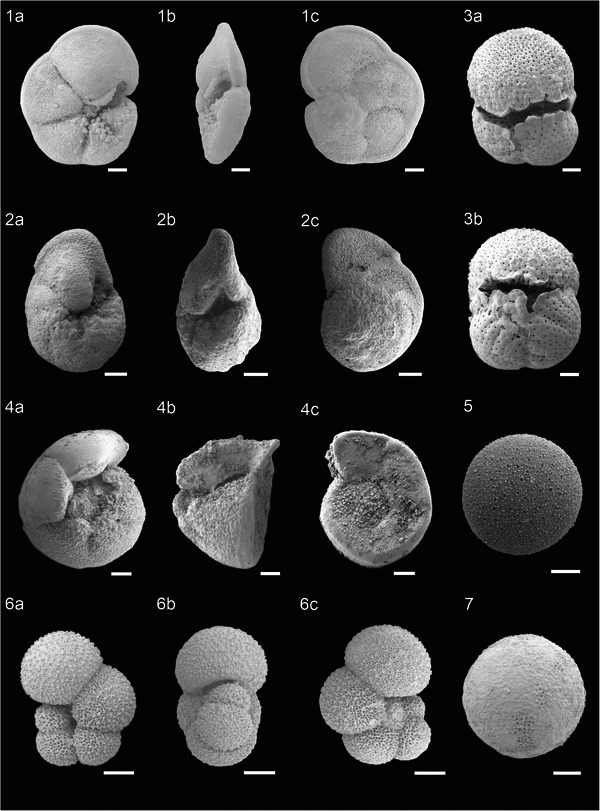
**1** *Gioborotalia menardii*, Sample U1498A 1R‐2W, 2‐4. a) umbilical view; b) lateral view; c) spiral view. **2** *Globorotalia tumida*, Sample U1498A 1R‐1W, 28‐30. a) umbilical view; b) lateral view; c) spiral view. **3** *Sphaeroidinella dehiscens*, Sample U1498A 1R‐1W, 58‐60. a) umbilical view; b) spiral view. **4** *Globorotalia truncatulinoides*, Sample U1498A 1R‐3W, 0‐3. a) umbilical view; b) lateral view; c) spiral view. **5** *Orbulina universa* found in pelagic sediments, Sample U1498A 1R‐4W, 61‐63. **6** *Globigerinella siphonifera*, Sample U1498A 1R‐2W, 2‐4. a) umbilical view; b) lateral view; c) spiral view. **7** *Orbulina universa* found in serpentinite nud deposits, Sample U1498A 4R‐1W, 82‐84. All scale bars are 100 µm except where otherwise stated. All specimens were imaged at 5 kv and with external secondary electron (SE) detector unless otherwise noted.

**Plate 4:**
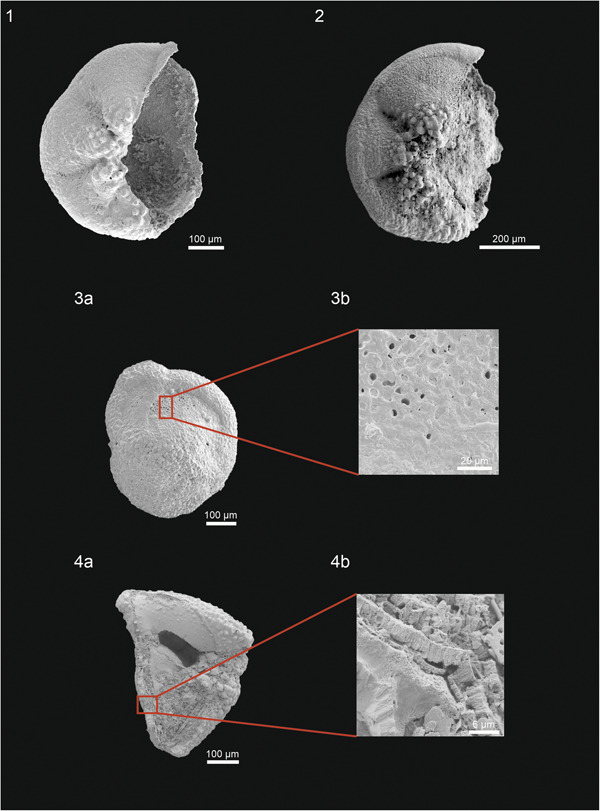
**1** specimen of *Globorotalia* showing overgrowth and broken test, Sample U1498A 1R‐1W, 6‐8 (inLens). **2** specimen of *Globorotalia* with overgrowth and broken test, Sample U1498A 1R‐2W, 89‐91. **3** a) overgrowth and pore widening on *Globororotalia* specimen; b) close‐up of the enlarged pores (inLens); Sample U1498A 1R‐4W, 61‐63. **4** a) dissolved test and keel of *Globorotalia truncatulinoides*, b) close‐up of the keel area; Sample U1498A 1R‐1W, 28‐30. a) umbilical view; b) lateral view. All specimens were imaged at 5 kv and with external secondary electron (SE) detector unless otherwise noted.

**Plate 5:**
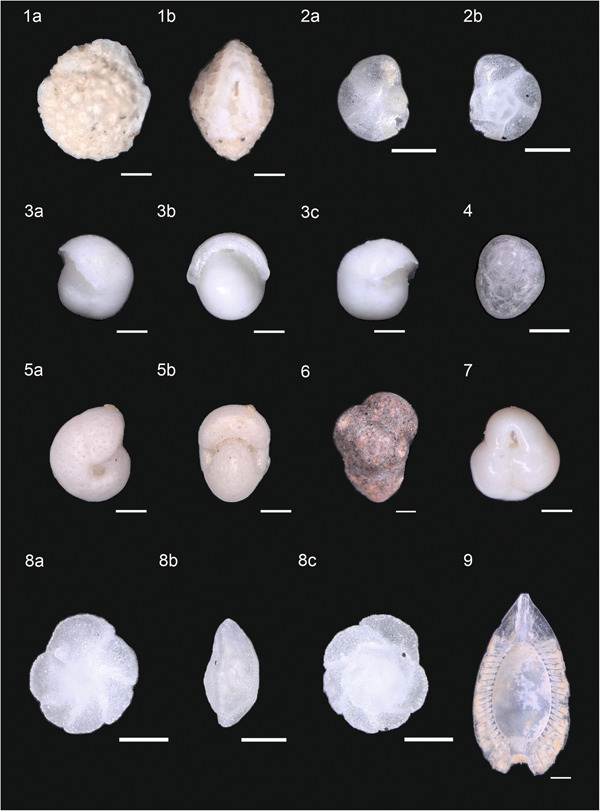
**1 a‐b** *Favocassidulina favus*, Sample U1498A 1R‐1W, 6‐8. **2 a‐b** *Epistominella exigua*, Sample U1498A 1R‐AW, 120‐122. **3 a‐c** *Pullenia bulloides*, Sample U1498A 1R‐1W, 6‐8. **4** *Rosalina bradyi*, Sample U1498A 1R‐1W, 58‐60. **5 a‐b** *Melonis pompilioides*, Sample U1498A 1R‐3W, 0‐3. **6** *Eggerella bradyi*, Sample U1498A 1R‐4W, 148‐150. **7** *Globocassidulina subglobosa*, Sample U1498A 1R‐3W, 29‐31. **8 a‐c** *Nuttallides umbonifer*, Sample U1498A 1R‐1W, 6‐8. **9** *Fissurina formosa*, Sample U1498A 1R‐4W, 148‐150. All scale bars are 100 µm except where otherwise stated.

**Plate 6:**
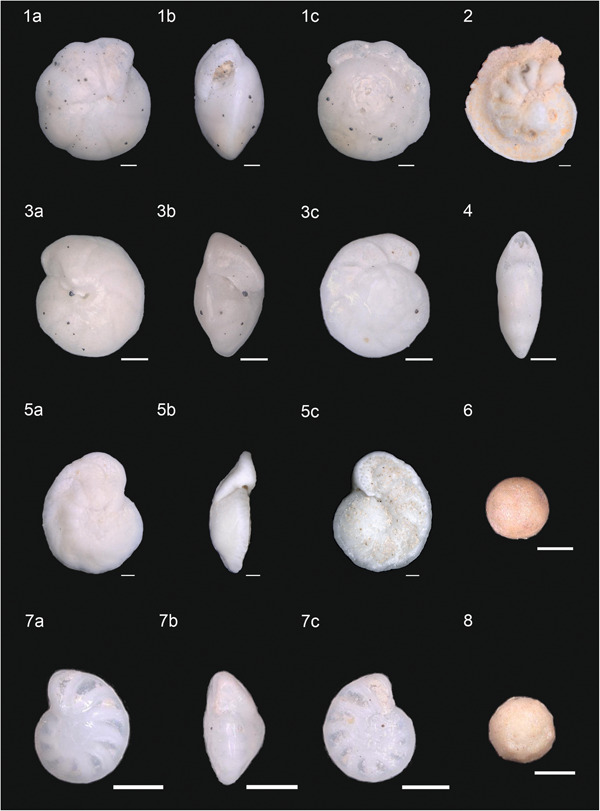
**1 a‐c** *Oridorsalis umbonatus*, Sample U1498A 1R‐1W, 6‐8. **2** *Laticarinina pauperata*, Sample U1498A 1R‐1W, 6‐8. **3 a‐c** *Hansenisca soldanii*, Sample U1498A 1R‐1W, 6‐8. **4** *Pleurostomella acuta*, Sample U1498A 13R‐2W, 15‐17. **5 a‐c** *Cibicidoides wuellestorfi*, Sample U1498A 1R‐3W, 0‐3. **6** *Thurammina albicans*, Sample U1498A 1R‐3W, 61‐ 63. **7 a‐c** *Cibicidoides mundulus*, Sample U1498A 1R‐3W, 61‐63. **8** *Thurammina papillata*, Sample U1498A 1R‐3W, 61‐63. All scale bars are 100 µm except where otherwise stated.

**Plate 7:**
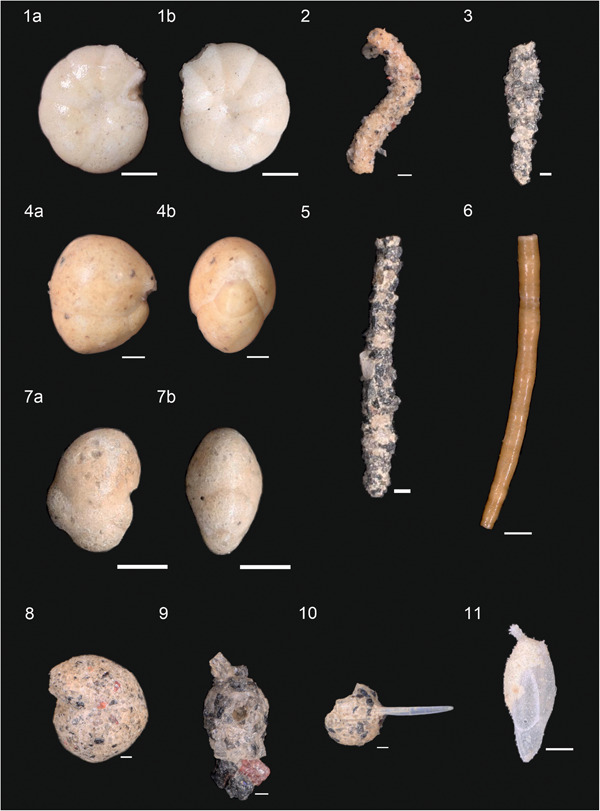
**1 a‐b** *Cyclammina trullissata*, Sample U1498A 1R‐1W, 58‐60. **2** *Testulosiphon indivisus*, Sample U1498A 1R‐3W, 61‐63. **3** *Psammosiphonella discreta*, Sample U1498A 1R‐2W, 2‐4. **4 a‐b** *Buzasina galeata*, Sample U1498A 1R‐1W, 58‐60. **5** *Rhabdammina abyssorum*, Sample U1498A 1R‐3W, 61‐63. **6** Nodellum membranaceum, Sample U1498A 1R‐3W, 61‐63. **7 a‐b** *Buzasina ringens*, Sample U1498A 1R‐1W, 6‐8. **8** *Cribrostornoides subglobosus*, Sample U1498A 1R‐1W, 6‐8. **9** *Reophax subfusiformis*, Sample U1498A 1R‐2W, 89‐91. **10** *Psammosphaera fusca*, Sample 1R‐1W, 58‐60. **11** *Francuscia extensa*, Sample 1R‐1W, 6‐8. All scale bars are 100 µm except where otherwise stated.

A total of 47 samples were collected at various intervals from Site U1498A and prepared for planktonic and benthic foraminifera analysis. The sediment was oven‐dried overnight at 40°C. Subsequently, samples were soaked in distilled water and washed through 500, 250, 125 and 63 µm sieves. Once dried at 40–50°C, the sediment was stored in labelled glass vials (Haynes, 1981; Snyder and Huber, 1996). After preparation, planktonic and benthic foraminifera were hand‐picked in the 500, 250 and 125 µm size fractions using a ZEISS DISCOVERY.V8 stereo microscope. Picked planktonic and benthic taxa were imaged using a ZEISS DSM 982 (Gemini) scanning electron microscope (SEM) and VXH‐6000 Keyence digital microscope. The SEM was also used to better evaluate the state of preservation. Specimens of the main planktonic foraminifera species and benthic taxa are shown in Plates 1–7 (see Appendix 1 and 2).

### Taxonomic remarks

The taxonomic identification of planktonic foraminifera largely derives from Blow (1969), Postuma (1971), Bylinskaya (2004), Kennett and Srinivasan (1983), Bolli and Saunders (1985), Chaisson and Leckie (1993), Loeblich and Tappan (1994), Weiner et al. (2015) and Wade et al. (2018). The taxonomy of benthic foraminifera is based on Morkhoven et al. (1986), Loeblich and Tappan (1988, 1994), Jones (1994) and Holbourn et al. (2013).

*Globigerinoides ruber* (white) represents several morphotypes (Jayan et al., 2021). Those morphological variants are separated for palaeoecological investigations into *G*. *ruber* sensu strictu (s.s.) and *G*. *ruber* sensu lato (s.l.) based on specific taxonomic criteria and stable isotopic composition (Wang, 2000; Numberger et al., 2009). In this study, we applied the taxonomic concept of Wang (2000) to differentiate the morphotypes. Specifically, all the specimens possessing three subspherical chambers in the last whorl, which are symmetrical over the previous sutures and with a wide, high‐arched aperture, were identified as *G*. *ruber* s.s. Conversely, individuals characterised by tighter tests with compressed, flattened and asymmetrical final chambers, as well as a relatively small aperture over the suture, were regarded as *G*. *ruber* s.l.

*Trilobatus sacculifer* plexus includes the four morphotypes *T*. s*acculifer* (Brady, 1877), *T*. *quadrilobatus* (d'Orbigny, 1846), *T*. *immaturus* (LeRoy, 1939) and *T*. *trilobus* (Reuss, 1850). We differentiated the morphological variants based on Poole and Wade (2019). In our analysis, we did not separate *T*. *immaturus* from *T*. *trilobus* as several individuals showed intergradational characteristics between the two morphotypes, making the differentiation problematic and inconsistent. The presence of the two morphotypes at the study site is documented in Plate 2 (see Appendix 1).

Several specimens exhibiting intermediate characteristics between *G*. *rubescens* and *G*. *woodi* were indicated as *Globoturborotalita* spp.

Planktonic species were separated into mixed layer and thermocline dwellers based on Chaisson (1995), Field (2004), Aze et al. (2011), Rebotim et al. (2017), Schiebel and Hemleben (2017), Pearson and Kucera (2018) and Jayan et al. (2021). Mixed layer taxa refer to species living in the upper portion of the ocean in which temperature and salinity values are relatively uniform. The thickness of this layer is usually 100–200 m. Thermocline dwellers include taxa that can be found within the thermocline, a transition layer below the mixed layer and above the deep water (>1000 m), within which temperature considerably changes with depth.

Tubular‐shaped benthic foraminifera (e.g. *Rhabdammina*, *Psammosiphonella*) were largely fragmented. To obtain a semi‐quantitative evaluation of the tubular individuals, fragments smaller than 1 mm were combined to reach a length of *c*. 1 mm. Larger fragments (>1 mm) were considered as a complete specimen following Hess (1998).

### Statistical analyses and ordination

At least 300 specimens per sample (when possible) were collected and identified to obtain a reliable statistical number for analysing the foraminiferal assemblages.

Overall, benthic foraminifera represent a minor component of the assemblage with discontinuous occurrences within the stratigraphic sequence. Thus, we only describe the benthic faunal composition, the absolute count of benthic species, and the ratio of epifaunal/infaunal forms (Tables S2, S4 and S7). All statistical evaluations and multivariate analyses were solely based on planktonic foraminifera.

**Figure 4:**
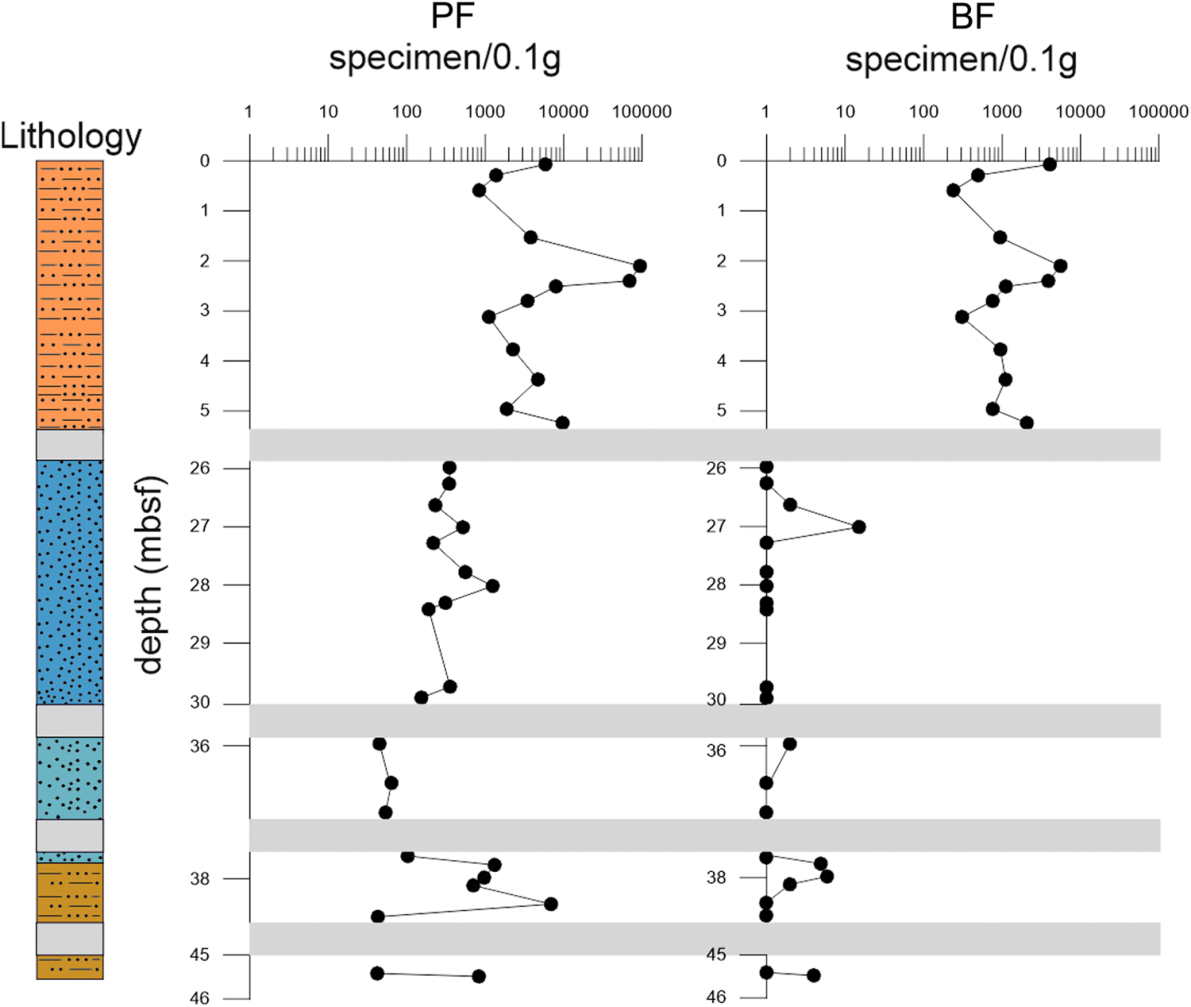
Absolute abundance of planktonic (PF) and benthic (BF) specimens (*x*‐axis) against depth (*y*‐axis). Grey bars: intervals with poor recovery and/or absence of suitable lithology for micropalaeontological investigations. Note the differing resolutions of the *x*‐axis. Barren samples show a value of 1 to be represented in a logarithmic scale. [Color figure can be viewed at wileyonlinelibrary.com]

**Figure 5:**
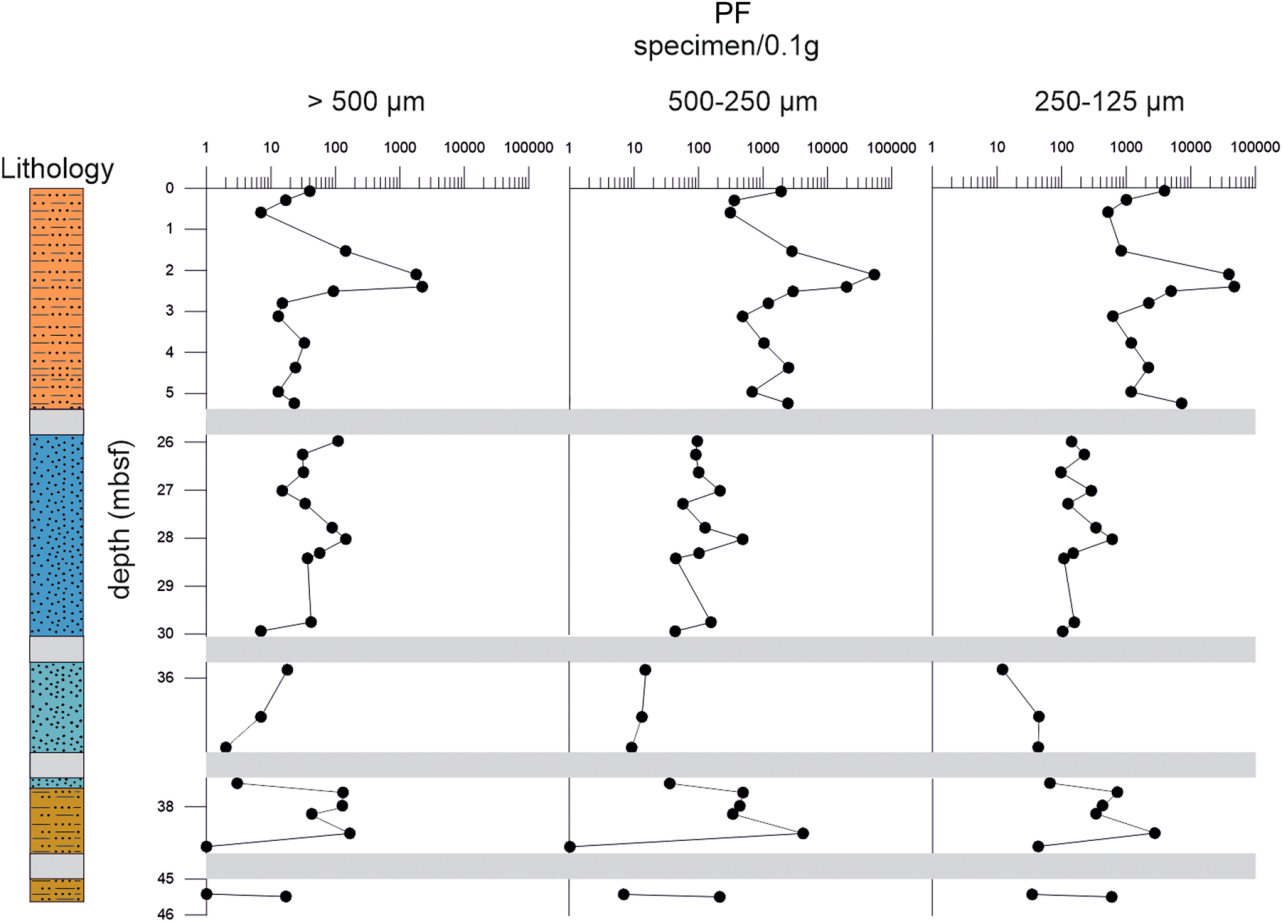
Absolute abundance of planktonic (PF) specimens (*x*‐axis) against depth (*y*‐axis) in each size fraction (>500, 500–250, 250–125 µm). Grey bars: intervals with poor recovery and/or absence of suitable lithology for micropalaeontological investigations. Note the differing resolutions of the *x*‐axis. Barren samples show a value of 1 to allow representation on a logarithmic scale. [Color figure can be viewed at wileyonlinelibrary.com]

The total number of foraminiferal individuals in the sediment was calculated considering the number of specimens counted in each sample and the number of aliquots (splits) used (Tables S3 and S4; Figures 4 and 5). Planktonic relative species abundances are determined as a percentage of the total count (Table S5; Figure 8).

**Figure 6:**
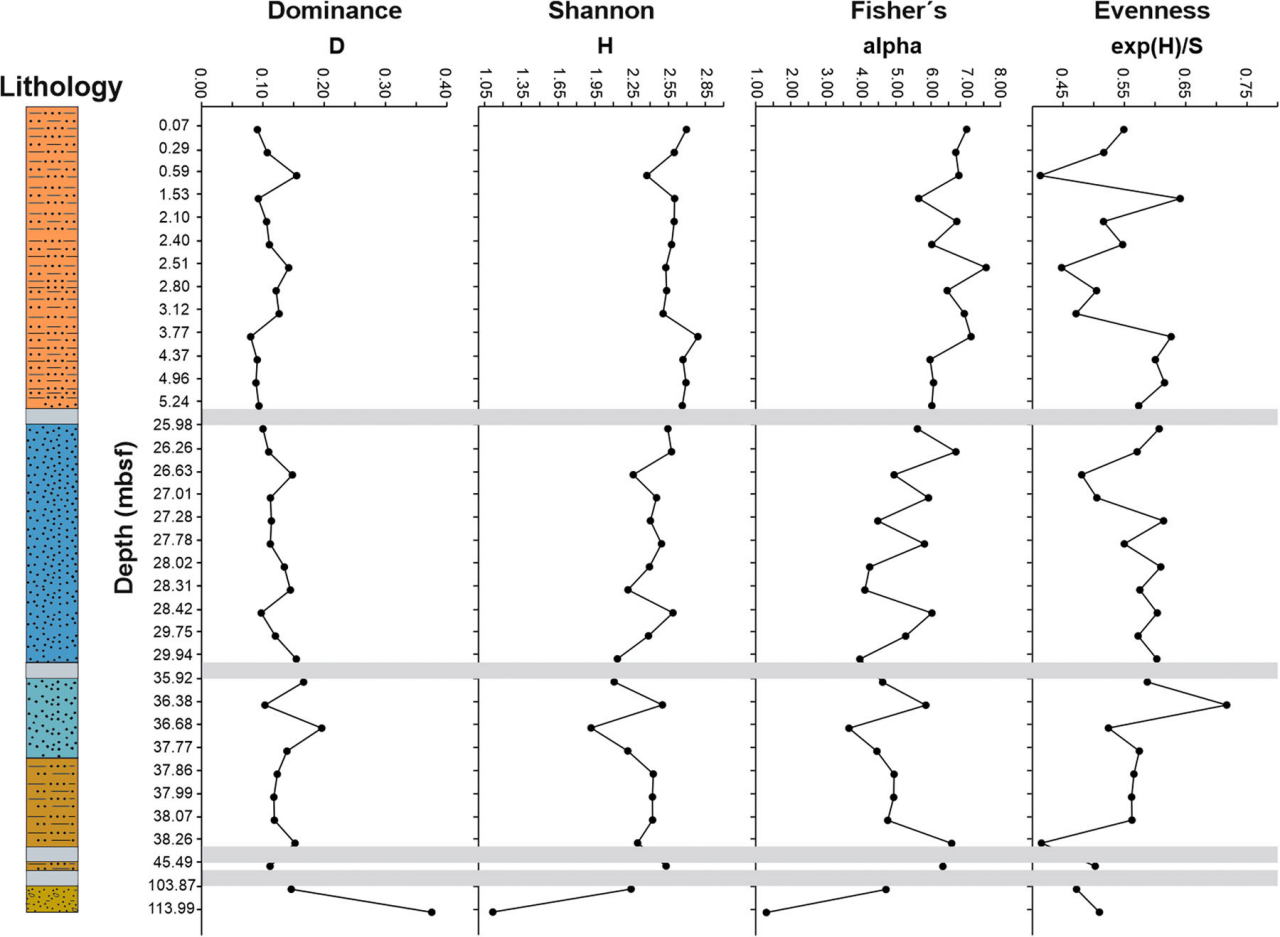
Diversity indices against depth (mbsf) calculated for planktonic foraminifera. The grey boxes represent intervals with poor recovery and/or absence of suitable lithology for micropalaeontological investigations. Note the different scales on the *x*‐axis. [Color figure can be viewed at wileyonlinelibrary.com]

**Figure 7:**
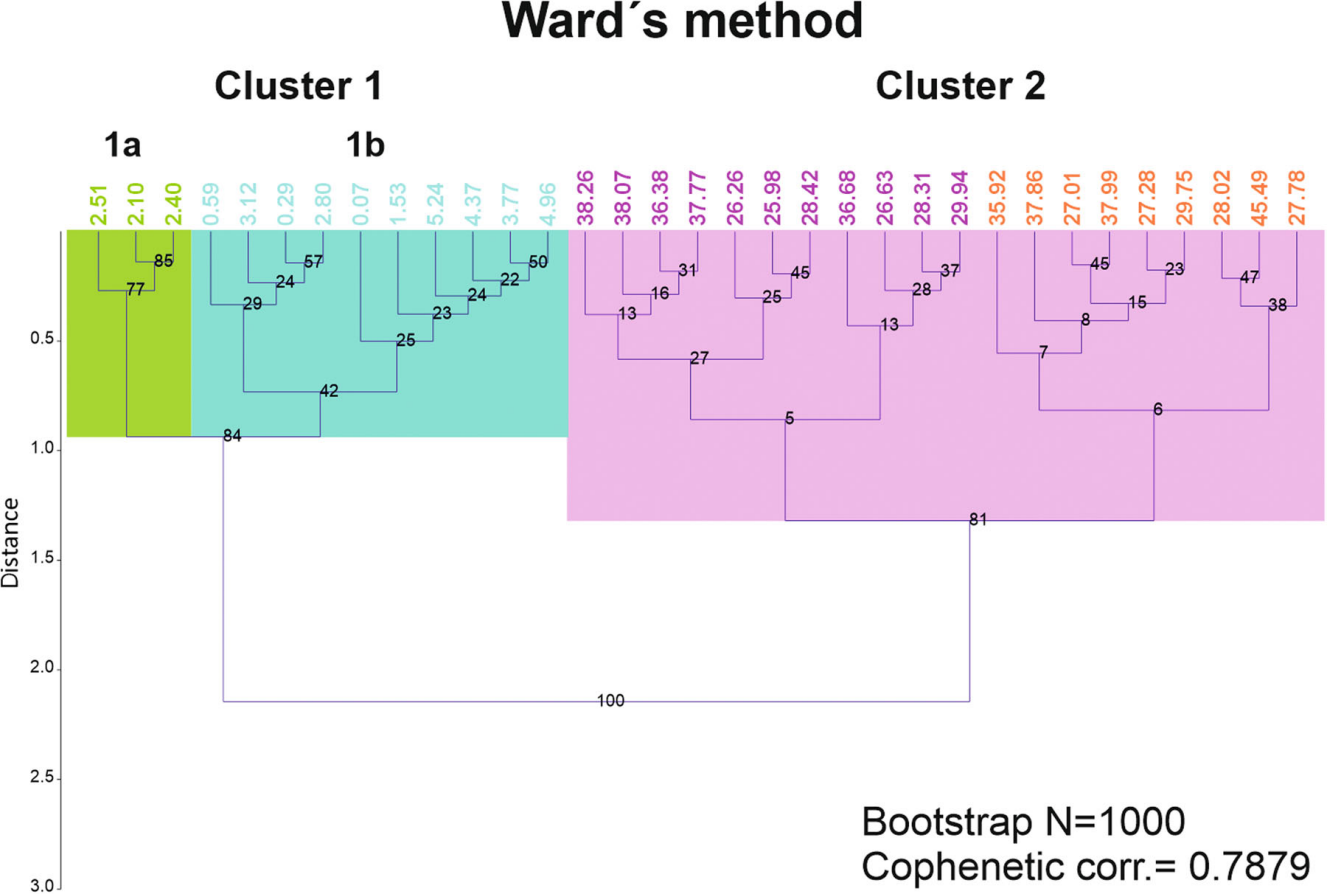
Dendrogram of the cluster analysis using Ward's method for planktonic foraminifera. Green box = subcluster 1a; blue box = subcluster 1b; pink box = cluster 2. Note that the orange and light purple colours were used to indicate the average depth of the samples belonging to the evaluated subclusters 2a and 2b. [Color figure can be viewed at wileyonlinelibrary.com]

Diversity indices Shannon index (H′) and Fisher's alpha index, as well as Evenness (exp(H)/S) and Dominance (D), were calculated using the number of planktonic individuals identified per sample (Table S6; Figure 6; Hammer and Harper, 2006). Samples containing a very low number of specimens (fewer than 90 specimens) were excluded from the calculations. Multivariate statistics and ordination methods (SIMPER, cluster analysis, principal component analysis [PCA] and non‐metric multidimensional scaling [nMDS]) were performed on planktonic foraminiferal assemblages using the software PAST (version 4.09) (Hammer et al., 2001). Arcsine root transformation (e.g. Sokal and Rohlf, 1995; Auer et al., 2019) on planktonic foraminifera relative abundances (Table S5) was computed before the multivariate statistics to exclude problems derived from a possible non‐normal distribution (Auer et al., 2019). Cluster analysis was performed using Ward's method (Ward, 1963), with the Euclidean similarity index (Figure 7) and unweighted paired group (UPGMA) (Sokal and Michener, 1958), using the Bray–Curtis similarity index (Fig. S1). We used Ward's method, with a cut‐off similarity of <0.70, to delineate clusters for subsequent interpretation (Figure 7). Bootstrapping (N = 1000) was applied to cluster analysis to examine the plausibility of the outcomes (Auer et al., 2019). Furthermore, we re‐ran the clustering, excluding the biostratigraphically restricted markers in the studied time interval, to test their effect on cluster distribution. We note that the sample distribution within the clusters was not markedly affected by excluding these taxa.

PCA using Bray–Curtis similarity was conducted as it enables variables (components) representing the maximum amount of variance of a multidimensional data set to be obtained, as well as validating the results of the cluster analysis (Fig. S2). Non‐metric multidimensional scaling (nMDS) with Bray–Curtis similarity (Fig. S3) was performed to test the validity of the PCA and cluster analysis results. Similarity percentage (SIMPER) analysis (Bray–Curtis similarity) was computed to assess the contribution of each species to the clusters (Table S5). For statistical analyses (e.g. diversity indices), we excluded samples with very low abundances (fewer than 90 specimens) of planktonic foraminifera and barren samples (Table S1). Moreover, indeterminate specimens were not considered for the multivariate statistics and the calculation of the diversity indices.

For multivariate analyses and relative abundance estimations, we grouped all *Trilobatus* morphotypes as *Trilobatus* spp. and all globorotalids as *Globorotalia* spp. The morphospecies of the *Trilobatus* group are considered to be one biological species, as demonstrated by molecular genetic analyses and culture experiments on extant specimens (Hemleben et al., 1987; André et al., 2013). Several samples within the pelagic deposit showed that individuals of *Globorotalia* (mainly subgenera *Globorotalia* and *Menardella*) were partially broken or fragmented. Therefore, only specimens with more than 50% of their shells preserved were counted as complete individuals. Furthermore, all recorded *Globorotalia* species showed the same trend in abundance (Table S5). Thus, grouping them does not affect the interpretation of the data.

*Globigerinoides ruber* s.s. and s.l. were consistently differentiated in our record (see Table S1). As the two morphotypes reflect different oceanographic conditions such as productivity and water stratification, they were treated as separate data entries in the statistical analyses.

## Results

A total of 11 473 foraminiferal individuals were picked and identified, of which 10 195 are planktonic, and 1278 belong to the benthic group. Planktonic foraminifera are represented by 50 species of 22 genera. Diversity of the benthic foraminifera is higher, with 67 genera and 99 identified species.

### Foraminiferal absolute abundances

Overall, planktonic foraminifera range between 0 and 94 363 (mean: 4865) specimens/0.1 g of sediment, while benthic foraminifera range from 0 to 5578 (mean: 487) specimens/0.1 g (Figure 4; Tables S3 and S4). Planktonic foraminifera exhibit the highest number of specimens (mean: 15 898; 841–94 363 specimens/0.1 g) within the pelagic deposits of Unit I (0.06–4.38 mbsf), with a peak in abundance in Sample 1R‐2W, 59–61 (2.10 mbsf). The total number of planktonic individuals decreases in the serpentinite mud deposits of Units III and IV (25.80–45.51 mbsf) with values from 42 to 6941 (mean: 709) specimens/0.1 g. Here, the lowest values are recorded between samples 5R‐1W 31‐33 cm and 5R‐2W 36‐38 cm (35.92–36.68 mbsf) and between samples 5R‐CC W 6‐8 and 6R‐CC W 1‐3 (38.39–45.42 mbsf).

Benthic foraminifera show a similar pattern, exhibiting values from 240 to 5578 (mean: 1715) specimens/0.1 g for Unit I and only 0 to 15 (mean: 2) specimens/0.1 g for Units III–IV, where benthic specimens are extremely scarce. In the lower part of the sequence (74.75–171.94 mbsf; Units V–VI), only very few samples contain a fair amount of planktonic and benthic foraminifera with values ranging between 0 and 6161 (mean: 531) and from 0 to 261 (mean: 45) specimens/0.1 g, respectively. The above‐mentioned basal part of the stratigraphic section was not included in the graphic representation as the high number of barren samples, the poor core recovery and the discontinuous sampling for this interval would have resulted in a significant difference in the resolution as well as an excessive number of gaps in Figure 4. Nevertheless, all data are available in Tables S3 and S4.

The absolute abundances of planktonic foraminifera were also evaluated in each analysed size fraction (>500, >250, >125 µm) (Figure 5; Table S3). The graph shows the absolute abundances for the size fractions (>500, 250–500, 250–125 µm) and displays similar trends. Absolute abundances show the highest values in the pelagic part of the stratigraphic sequence, ranging from 7 to 2221 (mean: 341), 311 to 53 661 (mean: 6941), and 523 to 47 184 (mean: 8616) specimens/0.1 g for >500, 500–250 and 250–125 µm, respectively. Abundance values decrease within the serpentinite mud units from 0 to 167 (mean: 51), 0 to 4050 (mean: 323), 12 to 2724 (mean: 335) specimens/0.1 g for 500, 500–250 and 250–125 µm, correspondingly. The forearc deposits show absolute abundances of 0 to 33 (mean: 3) specimens/0.1 g for the >500 µm size fraction, 0 to 245 (mean: 21) specimens/0.1 g, and 0 to 5916 (mean: 507) specimens/0.1 g for the 500–250 and 250–125 µm size fractions, respectively. As in Figure 4, absolute abundance data for the forearc material are not included in Figure 5. Nevertheless, data are displayed in Table S3. The comparison between different size fractions indicates that planktonic foraminifera are less abundant in the >500 µm fraction than in 500–250 µm and 250–125 µm size fractions. The highest planktonic foraminifera abundances for the >500 µm fraction were recorded between samples 1R‐2W 59–61 and 1R‐3W 1–3 (2.10–2.51 mbsf).

### Planktonic foraminifera

#### Preservation and reworking

The preservation of planktonic foraminifera is considerably variable at Site U1498A (Table S1). The pelagic sediments at the top of the sequence (Unit I; 0–5.40 mbsf) contain moderately to well‐preserved foraminiferal assemblages with individuals generally identifiable at the species level. Foraminiferal tests vary from white to opaque and brown, with some individuals also showing etched or broken shells. *Globorotalia* specimens were particularly affected by dissolution, exhibiting partial damage and fragmentation of their tests in several samples. Moreover, calcite overgrowth and pore widening were also visible on some of the foraminiferal tests. Downhole (Units III–VI; 29.95–172.05 mbsf), some planktonic foraminifera appear moderately etched and/or slightly compressed. Furthermore, sediment grains frequently covered the apertures of the specimens, and a confident identification was not always feasible. Regardless, foraminiferal tests range from white to opaque, and no fragments were observed in this part of the sequence.

A high number of indeterminate individuals occurred in several samples from the serpentinite mud deposits. Indeterminate specimens are whole test, sometimes only partially broken, compressed, altered and often completely covered in serpentinite matrix with no visible apertures.

Reworked planktonic foraminifera were explicitly noted in the serpentinite mud deposits (Units III–IV) and the forearc sediments of Unit VI (Table S1). The discrimination of reworked taxa was not based on colour or differences in preservation, as the specimens were often covered in matrix, mostly exhibiting the colour of the sediment they were contained in or filled with (from greenish to brownish).

A confident evaluation of the reworked species was obtained in the previous planktonic foraminiferal and calcareous nannofossil integrated biostratigraphic study conducted on samples from the same IODP site, U1498A (see Del Gaudio et al., 2022).

Serpentinite mudflow units mainly exhibited Miocene to Pliocene reworked forms such as *Globoturborotalita woodi*, *G. decoraperta*, *G. nepenthes*, *Globigerinoides bollii*, *Globigerinella obesa* and *Pulleniatina praecursor*, with scarce specimens of *Globigerinoides subquadratus*. The forearc sediments contain reworked Miocene (Burdigalian‐Tortonian) faunal elements (e.g. *Globigerinoides subquadratus*, *Globoturborotalita druryi* and *Sphaeroidinellopsis disjuncta*). Previous biostratigraphic analysis detected no reworking within the pelagic layers (Unit I) (Del Gaudio et al., 2022).

#### Planktonic foraminifera diversity

The minimum number of species recorded in a single sample is six (Sample 13R‐2W, 15–17 cm), while the maximum number is 28 (Sample 1R‐3W, 0–3). Shannon (H) and Fisher's alpha indices show similar trends (Figure 6; Table S6). High diversity occurs within the pelagic mud deposits with average values of 2.60 and 6.55 for Shannon (H) and Fisher's alpha, respectively. The diversity slightly decreases in the serpentinite mud deposits (Units III–IV) with average values of 2.36 (Shannon) and 5.17 (Fisher's alpha) and drop within the forearc deposits to 1.68 and 3.01 for Shannon and Fisher's alpha, respectively. Dominance (D) shows higher values in the forearc deposits (average: 0.26) compared with the pelagic and serpentinite mudflow units (average: 0.11 and 0.13, respectively). The Evenness index (exp(H)/S) exhibits close average values of 0.54 and 0.56 for the pelagic and serpentinite mud deposits but decrease to 0.49 within the forearc sediments. Two positive and two negative peaks were observed within the pelagic cover and the serpentinite mud volcano layers, respectively (Figure 6). The negative peak in the pelagic unit was detected in Sample 1R‐1W, 58–60 (0.59 mbsf), whereas the positive peak was recorded in Sample 1R‐2W, 2–4 (1.53 mbsf). The two samples contained *G*. *ruber* s.l., which shows its highest number of specimens (83) in Sample 1R‐1W, 58–60, and the lowest number of individuals (37) in Sample 1R‐2W, 2–4 within the pelagic interval. Moreover, two additional peaks in evenness (one positive in Sample 5R‐2W, 6–8, and one negative in Sample 5R‐3W, 50–52) were detected in the serpentinite mud deposits. In both samples, *G. ruber* s.s. and *Globigerinita glutinata* were present. Both show a low number of individuals in Sample 5R‐2W, 6–8 (12 and 21) with similar values to the other species in the assemblage. Their number of specimens is very high in Sample 5R‐3W, 50–52 (62 and 58) compared with the other species.

#### Cluster analyses and ordination

Hierarchical clustering (Ward's method) resulted in two main clusters (Figure 7): cluster 1 consists of samples from the pelagic cover, whereas cluster 2 is composed of samples belonging to the serpentinite mud deposits. The separation of the clusters occurred at a cut‐off distance of ~1.4. The cophenetic correlation coefficient obtained with Ward's method is 0.79. Bootstrapping (N = 1000) validated the results. UPGMA using Bray–Curtis similarity (Fig. S1) also supported the separation of the dataset into two major clusters (cut‐off similarity >0.70; cophenetic correlation coefficient of 0.83). PCA and nMDS with Bray–Curtis similarity confirmed the two principal clusters (see Figs. S2 and S3).

The results of Ward's clustering further indicated that cluster 1 can be divided into two subclusters (1a and 1b) with a low cut‐off distance of 0.90 (Figure 7). However, UPGMA does not support the partition of cluster 1 in the two subclusters 1a and 1b (Fig. S1). PCA shows a distinct differentiation between the two subclusters and the nMDS plot, despite overlapping with one sample (1R‐2W 59–61; 2.10 mbsf), also supports subclusters 1a and 1b. Samples included in cluster 1a (Table S5) consist of a higher amount of coarser fraction, almost entirely composed of planktonic foraminifera (very few serpentinite clasts) and with the highest number of planktonic foraminifera in the larger size fraction within the pelagic deposit (>500 µm, 500–250 µm). SIMPER analysis indicates that subclusters 1a and 1b were largely separated based on the abundances of *Globigerinoides conglobatus* and *Orbulina universa* (Table S5) with a taxon contribution >11% (cumulative 23.46%). Based on these results, we consider the separation of the above‐mentioned subclusters to be robust. The subclusters for cluster 2 obtained with Ward's method (Figure 7) were not supported by UPGMA analysis (Fig. S1). Furthermore, both PCA and nMDS plots show a distinct overlapping of subclusters 2a and 2b (Figs. S2 and S3). Thus, we excluded these subclusters and considered all samples to belong to cluster 2.

Cluster analysis was also computed by excluding the stratigraphic marker species (Del Gaudio et al. 2022). However, similar results were attained with and without biostratigraphically relevant taxa.

**Figure 8:**
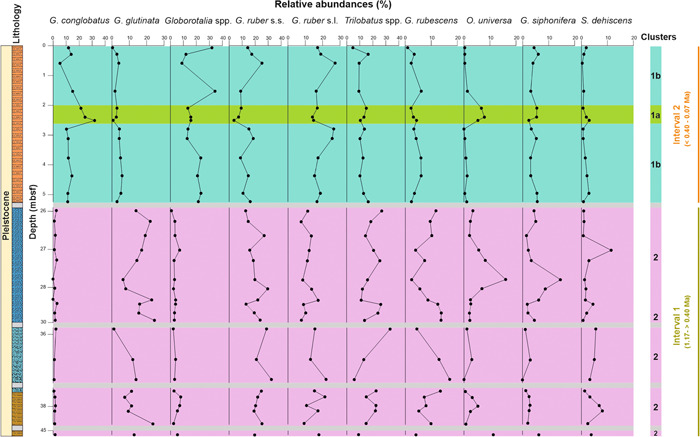
Relative abundances (%) of the most abundant planktonic species at Site U1498A. Note the different scales at the *x*‐axis. Grey boxes = intervals with poor recovery and/or absence of suitable lithology for micropalaeontological investigations. 1a = subcluster 1a; 1b = subcluster 1b; 2 = cluster 2. Full binomial nomenclature of the represented taxa/species groups as follows: (from left to right) *Globigerinoides conglobatus*, *Globigerinita glutinata*, *Globorotalia* spp., *Globigerinoides*. *ruber* s.s., *Globigerinoides*. *ruber* s.l., *Trilobatus* spp., *Globoturborotalita rubescens*, *Orbulina universa*, *Globigerinella siphonifera*, *Sphaeroidinella dehiscens*. Possible age ranges for the intervals are indicated in brackets. [Color figure can be viewed at wileyonlinelibrary.com]

**Figure 9:**
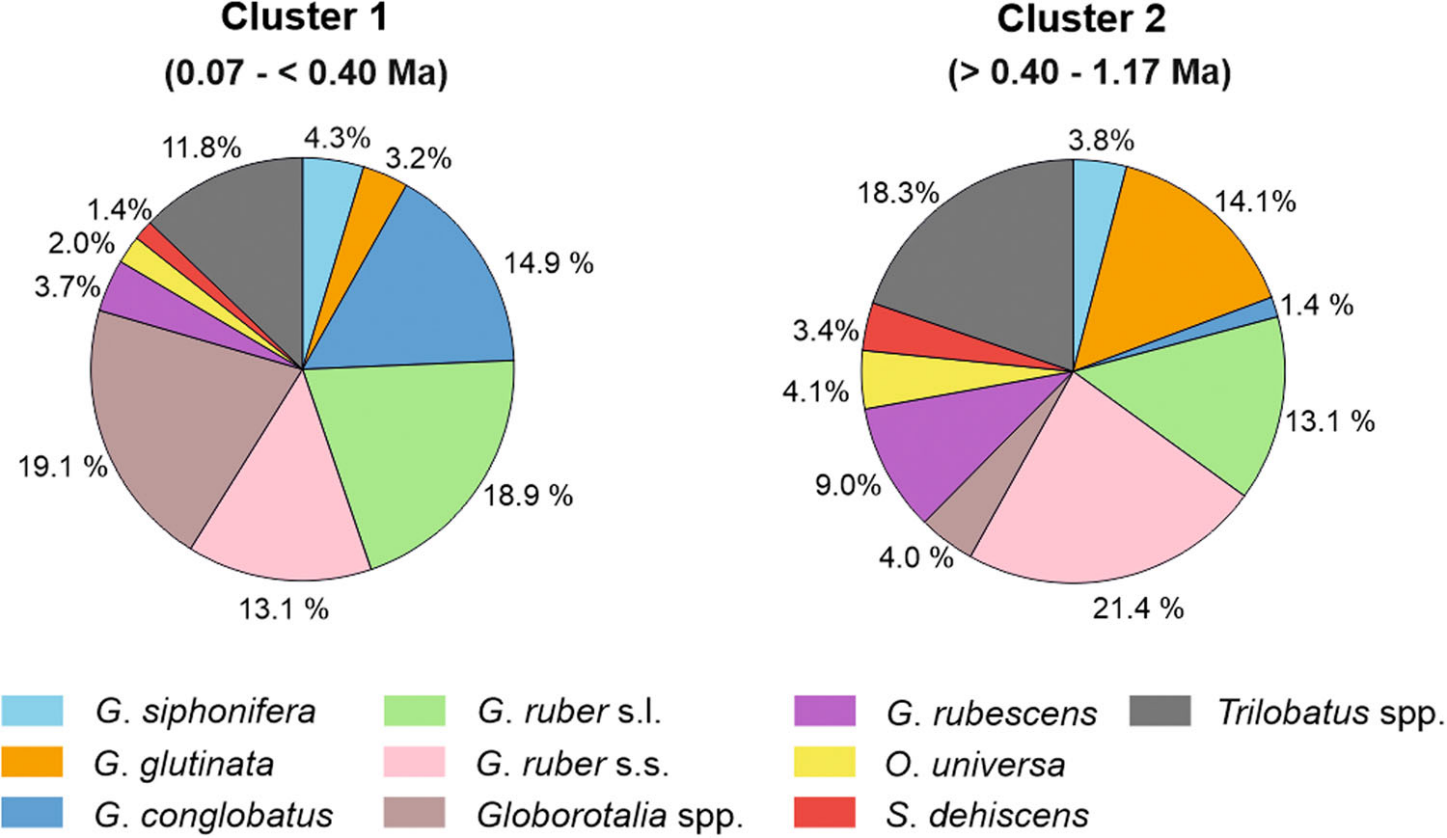
Average abundances (%) of the main species for clusters 1 and 2 obtained from SIMPER analysis (contribution >2%). Possible age ranges for the intervals are indicated in brackets. [Color figure can be viewed at wileyonlinelibrary.com]

SIMPER analysis was applied to clusters 1 and 2 (Table S5), calculating the dissimilarity between the two groups in terms of assemblage composition (%). The most important taxa contributing to the separation of clusters 1 and 2 (contribution >2%) are *Globorotalia* spp., *G*. *conglobatus* (individual contribution >15%) as well as *Globigerinita glutinata*, *G*. *ruber* s.s., *Trilobatus* spp., *G*. *ruber* s.l., and *Globoturborotalita rubescens* (contribution between 13 and 6%). Those species overall accounted for 79% of the variance. Other species include *O*. *universa*, *Globigerinella siphonifera* and *Sphaeroidinella dehiscens* (contribution between 2.7 and 4.25%), contributing 10% of the dissimilarity, cumulatively. The relative abundance trends and average abundances (%) of the above‐mentioned most important species, according to SIMPER analysis, are shown in Figures 8 and 9. Data are also available in Table S5.

Cluster 1 includes all samples from the pelagic cover (0.06–5.24 mbsf). The most abundant taxa are *G*. *ruber* (average 32% with *G*. *ruber* s.s. = 13.1% and *G. ruber* s.l. = 18.9%), *Globorotalia* spp. (19.1%), *G*. *conglobatus* (14.9%) and *Trilobatus* spp. (11.8%). Other species include *G*. *siphonifera* (4.3%), *G*. *rubescens* (3.7%), *G*. *glutinata* (3.2%), *G*. *bulloides* (3.0%), *O*. *universa* (2.0%) and *S*. *dehiscens* (1.4%). *Neogloboquadrina dutertrei* (0.8%), *Globigerinella calida* (0.6%), *Globigerinoides tenellus* (0.5%), *Pulleniatina obliquiloculata* (0.4%), *Candeina nitida* and *Beella digitata* (0.3%) were rare components of the assemblage. A peak in abundance of *G*. *conglobatus* and *O*. *universa* can be observed in the depth interval 2.10–2.51 mbsf.

Cluster 2 consists of samples representing the sandy and silty multicoloured serpentinite mud deposits of Units III and IV (25.80–45.51 mbsf). This group is defined by the high abundance of the *G. ruber* group (average 35%; with *G*. *ruber* s.s. = 21.4% and *G. ruber* s.l. = 13.1%), *Trilobatus* spp. (18.3%) and *G*. *glutinata* (14.1%). Other common taxa include *G*. *rubescens* (9%), *O*. *universa* (4.1%), *Globorotalia* spp. (4%), *G*. *siphonifera* (3.8%) and *S*. *dehiscens* (3.4%). Minor components of the assemblage are *Globigerina bulloides* (2.1%), *G*. *conglobatus* (1.4%) and *P*. *obliquiloculata* (1.1%).

By comparing the two clusters, it can be seen that in cluster 2, *G*. *ruber* s.s. shows higher abundance (21.4%) than *G*. *ruber* s.l. (13.1%). *G*. c*onglobatus* and *Globorotalia* spp. exhibit low relative abundances (1.4% and 4%, respectively) in cluster 2. Conversely, *G*. *glutinata* and *G. rubescens* greatly increased in the serpentinite mud deposits (Table S5). *Orbulina universa*, *Trilobatus* spp. and *S*. *dehiscens* show a moderate increase of 2.1, 6.5 and 2%, respectively, compared with cluster 1. The abundances of *G*. *bulloides* and *G*. *siphonifera* do not change considerably (Table S5).

PCA results (Figure S2) show that two variables explain 58% of the variance (PC1 = 46.48%; PC2 = 11.51%), with PC1 being the most important for separating the two clusters. An additional two variables showed values above the broken‐stick curve and must therefore be considered (PC3 = 8.89; PC4 = 7.00), highlighting the complexity of the dataset. Nevertheless, PC1 and PC2 separate clusters 1 and 2. Specifically, the plot shows that cluster 1 strongly depends on *G*. c*onglobatus*, *Globorotalia* spp., and to a lesser extent on *G*. *ruber* s.l. Cluster 2 is mainly characterised by *G*. *glutinata* and *G*. *rubescens*, and moderately by *G*. *ruber* s.s. and *Trilobatus* spp.

In terms of their water depth distribution (Table S5), cluster 1 has a dominance of mixed layer taxa (average 70.24%) over thermocline dwellers (29.83%). In cluster 2, mixed layer dwellers became even more abundant (81.72%), whereas thermocline taxa dropped to 18.28%.

In the lowermost part of the stratigraphic sequence, only a few samples within the forearc deposit show a reasonable number of planktonic foraminifera for statistical analysis (>90). Therefore, we excluded those samples from the multivariate analyses and subsequent data interpretation. Nevertheless, the main species within the assemblage are *Globoturborotalita woodi* (33.8%) and *Sphaeroidinellopsis seminulina* (22.8%). Other common species are *G*. *glutinata* (7.3%), *G*. *ruber* s.s. (6.8%), *Globoturborotalita decoraperta* and *Trilobatus* spp. (6.5%). The minor components of the assemblage and their percentages are available in Table S5.

### Benthic foraminifera

#### Preservation and reworking

Benthic foraminifera are generally well‐ to moderately well‐preserved (Table S2). Most specimens display white to brown tests with partial etching of the shell. Calcite overgrowth was also visible on individual specimens. Tubular foraminifera (e.g. *Rhabdammina*, *Psammosiphonella*) were mostly fragmented. No reworking of benthic forms was observed within the different lithologies.

### Assemblage data

Overall, benthic foraminifera represent a small component of the foraminifera fauna (Table S2, S4; Figure 4). Within the pelagic unit (0.06–5.24 mbsf), the benthic fauna shows high abundances of *Nuttallides umbonifer* (average 28.5%), *Globocassidulina subglobosa* (15.1%) and tube‐shaped agglutinated species such as *Psammosiphonella discreta*, *Rhabdammina abyssorum* and *Testulosiphon indivisus* (13.6%). Other common species include *Epistominella exigua* (5.4%), *Osangularielloides rugosus* (3.6%), *Oridorsalis umbonatus* (3.3%) and *Cribrostomoides subglobosus* (2.4%). *Bolivina* spp. and *Bulimina* spp. are rare components of the assemblage (0.2%). The epifaunal/infaunal ratio for the above‐mentioned depth interval (Table S7) indicates that epifaunal taxa prevailed over infaunal species (70.3% and 26.8%, respectively).

The number of benthic specimens drastically decreases in the serpentinite mudflow deposits (25.80–45.51 mbsf) where rare individuals of *Cibicidoides wuellerstorfi*, *G*. *subglobosa*, *N*. *umbonifer*, *Oolina* spp., *O*. *umbonatus* and *Triloculina* spp. were recorded (Table S2). Only a few samples within the forearc deposits (74.75–171.94) contain a suitable number (approximately 100) of benthic specimens (Table S2). The assemblage was characterised by common *N*. *umbonifer* (average 20.6%), *G*. *subglobosa* (19.7%), agglutinated tubular forms (10.0%), *O*. *umbonatus* (10.5%), *Hansenisca soldanii* (7.0%) and *Pullenia bulloides* (6.6%). Other taxa recorded were: *Pullenia quinqueloba* (5.7%), *Gyroidina umbonata* (5.3%), *E*. *exigua* (3.5%) and *Cibicidoides mundulus* (2.6%).

The infaunal group (average 39.9%) in the forearc material increased in abundance compared with the pelagic deposit of Unit I (Table S7). However, the epifaunal species remained the major constituents of the benthic assemblage (52.5%).

## Discussion

The foraminiferal assemblages in the stratigraphic sequence are dominated by planktonic foraminifera, indicating favourable conditions within the water column. Planktonic foraminifera show the highest absolute abundances in the pelagic unit (0–5.40 mbsf; Figure 4). Lower values were observed within the serpentinite mud deposits (25.80–45.51 mbsf), in which planktonic foraminifers are still the dominant component of the assemblage. The decrease in the absolute abundance detected in the mudflow deposits is possibly attributable to dilution. Similarly, benthic foraminifera are abundant in the pelagic sediment but extremely low in the serpentinite mud units. The outflow of serpentinite mud and the gas exhalations represent severe environmental stress, resulting in a hostile habitat for benthic communities. The low number of samples containing foraminifera and poor sediment recovery in the lower part of the section (74.75‐171.94 mbsf) did not allow a palaeoecological interpretation of the assemblage.

### Planktonic foraminifera

Planktonic foraminifera indicate tropical to subtropical conditions as the assemblage is predominantly composed of typical tropical–subtropical taxa such as *G*. *ruber*, *G*. *conglobatus*, *G*. *rubescens*, *Trilobatus* spp., *O*. *universa* and warm water globorotalids (e.g. *G*. *menardii*, *G*. *truncatulinoides*, *G*. *tumida*) (Bé and Tolderlund, 1971; Kennett and Srinivasan, 1983; Kucera 2007).

### Planktonic foraminifera diversity and reworking

The planktonic foraminiferal diversity exhibits low values in the forearc deposits (103.80–172.05 mbsf), whereas high diversity was recorded for the pelagic unit (0–5.40 mbsf) and the serpentinite mudflow sequence (25.80–45.51 mbsf) (Table S6). Dominance of planktonic foraminifera is low in the upper part of the sequence (0–45.51 mbsf) but increases within the forearc unit, where the assemblage contains high abundances of only two species (*G*. *woodi* and *S*. *seminulina*; Table S5).

Close average values of evenness were obtained for the pelagic and the serpentinite mud units (Table S6), suggesting that the species are equally distributed within the assemblage in both units. The negative and positive peaks in evenness within the pelagic cover correlate with the lowest and the highest number of specimens of *G*. *ruber* s.l. (Table S6), which is the most abundant species. A positive and negative peak were also observed in the serpentinite deposits. The lowest value of evenness in the sample coincides with the dominance of *G*. *glutinata* and *G*. *ruber* s.s.

Reworking processes in the Mariana forearc deposits were indicated by previous papers (Ciampo, 1992; Menapace et al., 2019; Del Gaudio et al., 2022). In this study, reworked taxa were observed in the serpentinite mud and forearc deposits. In particular, sediments from the serpentinite mud units contain predominantly reworked Pliocene taxa with very rare Miocene forms. Reworking of planktonic foraminifera may be attributed to mass movements of older material along the flank of the Fantangisña seamount or remobilisation from subsequent mudflow events. Moreover, the presence of Pliocene and Miocene reworked species could indicate the existence of older serpentinite mudflows.

### Palaeoecological reconstruction based on planktonic foraminifera

Based on cluster analyses, the planktonic assemblage can be grouped into two clusters (clusters 1 and 2), with cluster 1 split into two subclusters (see section ‘Cluster analyses and ordination’, above). Clustering is not affected by the stratigraphic distribution of the planktonic foraminifera species. Therefore, based on the cluster‐based grouping of samples with similar planktonic foraminifer assemblage composition, it is possible to ascribe similar environmental factors to the defined clusters. Planktonic foraminifera exhibit a vertical distribution within the water column (Bé, 1960; Chaisson, 1995; Douglas and Savin, 1978; Fairbanks et al., 1980; Keller, 1985; Gasperi and Kennett, 1993) that depends on several variables such as salinity, oxygen content, nutrients and the intensity of upwelling (Field, 2004; Kucera 2009; Rebotim et al., 2017; Lessa et al., 2020). Among them, an important parameter is the DOT, which represents the region in the water column situated beneath the mixed layer where a rapid vertical temperature change occurs (Kaiser et al., 2005). Shifts in the DOT are proven to be related to currents, winds, global climate changes, upper water temperature, upwelling and variation in marine productivity (Bé et al., 1985; Brasier, 1995; Chaisson, 1995; Andreasen and Ravelo,^1997^; Jian et al.,^2000^; Andruleit et al., 2008; Liu and Zhou, 2020). When the DOT deepens, the number of mixed layer taxa increases, whereas the abundance of the thermocline dwellers drops, and vice versa (Ravelo et al., 1990; Ravelo and Fairbanks, 1992; Jian et al., 2000). Our data indicate different DOT during the Early–Middle and Late Pleistocene, based on a clear stratigraphic separation of clusters 1 and 2 in the depth domain. Hence clusters 1 and 2 constitute different foraminiferal assemblages – or more precisely taphogroups – which denote stratigraphic intervals with a different DOT during the Pleistocene at Site U1498 (Figure 8–9). The following section will thus describe the two palaeoecological intervals as defined by cluster analysis in detail:

*Interval 1: Early–Middle Pleistocene transition (EMPT)*

The planktonic foraminifera assemblage of the serpentinite mud deposits (cluster 2) indicates a deep, stable and stratified water column with abundant mixed layer taxa (e.g. *G*. *ruber*, *G*. *conglobatus, Trilobatus* spp., *G*. *glutinata*, *G*. *rubescens*) (Chaisson, 1995; Aze et al., 2011). Specifically, the morphotype *G*. *ruber* s.s. and *Trilobatus* spp. are most abundant within the analysed planktonic community.

*Globigerinoides ruber* s.s. shows higher average abundances in this interval than *G*. *ruber* s.l. (Figures 8, 9). Stable isotope studies in the Pacific Ocean (Wang, 2000; Kawahata, 2005; Numberger et al., 2009) indicated a different calcification depth for G. *ruber* s.s. and s.l., with the latter calcifying deeper in the water column. Moreover, the habitat of the two morphotypes is related to oceanographic conditions such as productivity and stratification (Wang, 2000; Numberger et al., 2009). A study by Jayan et al. (2021) showed that the abundances of *G*. *ruber* morphotypes depend on the depth of the mixed layer, emphasising their use as a proxy for the water column structure. Specifically, *G*. *ruber* s.l. prefers lower sea surface temperature (SST) and less stratified water, whereas the abundance of *G*. *ruber* s.s. increases with higher SST and stronger stratification. The higher abundances of *G*. *ruber* s.s. support our interpretation of a deep and stable thermocline.

*Orbulina universa* is widely considered a mixed layer dweller (Spero, 1987; Chaisson, 1995; Seears et al., 2012). However, Vergnaud‐Grazzini (1976) reported *O*. *universa* as a sub‐thermocline species based on δ^13^C and δ^18^O values. In our record, *O*. *universa* shows the same abundance pattern of the main mixed layer taxa (Figure 8), which increases in abundance when the total number of thermocline dwellers drops. Thus, our data agree with the studies indicating that *O*. *universa* is a mixed layer dweller.

Interval 1 falls within the Early–Middle Pleistocene transition (EMPT), an event spanning from 1.4 to 0.4 Ma (Head and Gibbard, 2015), during which a change in the periodicity of the climatic cycles took place (Berger and Jansen, 1994). A persistent SST cooling trend was observed at low and high latitudes during the EMPT except for the Western Pacific Warm Pool, where the SST remained constant (de Garidel‐Thoron et al., 2005; Medina‐Elizalde and Lea, 2005; Clark et al., 2006) and the northwestern Pacific, affected by secular warming (Head and Gibbard, 2015). Radiolarian and planktonic foraminifer assemblages from North Pacific low latitudes (IODP Expedition 314–315; ODP Site 1144) indicated that warm and oligotrophic conditions persisted during most of the EMPT, between ~1.3 and 0.7 Ma (Wang et al., 2003; Zheng et al., 2005; Matsuzaki et al., 2015; Kubota et al., 2021), supporting the presence of a deep, stable thermocline. Similarly, calcareous nannofossil assemblages from Shatsky Rise (ODP Site 1209) suggest warm SSTs and stable conditions during the EMPT between ~ 1.2 and 0.62 Ma (Lupi et al., 2019).

*Interval 2: Middle–Late Pleistocene (post‐EMPT)*

Interval 2 corresponds to the planktonic foraminifera assemblage of the oxidised Middle to Late Pleistocene pelagic sediments summarised in cluster 1. Subcluster 1a includes three samples (2.10–2.51 mbsf; Figure 7) and its separation is mainly related to *G*. *conglobatus* and *O*. *universa* (Table S5), which show a peak in abundance between 2.10 and 2.51 mbsf (Figure 8). The highest absolute abundances of planktonic foraminifera in the coarser fraction (>500 µm) were recorded in this depth interval (Figure 5; Table S3), and the test size of *G*. *conglobatus* and *O*. *universa* is generally larger than 250 µm (Young et al., 2017). Samples corresponding to the afore‐mentioned depth interval (2.10–2.51 mbsf) are almost exclusively composed of foraminifera tests with very few ultramafic clasts, indicating less dilution from the mud and, thus, the predominance of pelagic sedimentation.

Compared with interval 1, a weaker and less stratified thermocline is indicated in interval 2 based on our interpretation of cluster 1. The shoaling of the thermocline is supported by a noticeable increase in thermocline dwellers (particularly globorotalids) and the corresponding reduction in the abundance of mixed layer taxa (Figure 9). Specifically, the relative abundances of *G*. *ruber*, *Trilobatus* spp., *G*. *rubescens* and *G*. *glutinata* dropped in this interval (Figure 8). In contrast to interval 1, *G*. *ruber* s.l. exhibit higher average abundances (Figures 8 and 9), possibly supporting the existence of a weaker thermocline.

Data from the nearby South China Sea and the western equatorial Pacific (Schmidt et al., 1993; Jian et al., 2000) showed that the thermocline gradually became shallower after ~0.7 Ma until MIS 6–5 (0.19–0.07 Ma) where the shallowest DOT was recorded, accompanied by a consistent reduction of the SSTs (Jian et al., 2000). SST reconstructions in the northwestern Pacific Ocean near Shatsky Rise (Ujiié, 2003 and LaRiviere et al., 2012; Sites 1208–1209) also indicated low SSTs during MIS 7–6, with the coolest temperatures recorded during MIS 6. Although our record is too coarse to observe a shallowing trend of the thermocline, we clearly see that planktonic foraminifera data indicate a weaker thermocline after 0.4 Ma. The shallowing of the thermocline of interval 2 may be linked to one of the low SST phases recorded in the previously discussed studies.

We therefore propose that the fluctuations of the DOT are related to phases of intensification and weakening of the NEC. Changes in the DOT related to eutrophication can be excluded as the site lies within the oligotrophic subtropical gyre, and the upwelling indicators (e.g. *G*. *bulloides*) exhibit low abundances within the entire stratigraphic section.

The strengthening of the modern NEC is linked to the ENSO (Qiu and Joyce, 1992; Kashino et al., 2009; An et al., 2018). During El Niño, the WC is greatly reduced, the NEC weakens and the thermocline shoals. Conversely, a strong WC is recorded during La Niña, when the intensity of the NEC noticeably increases and more warm oligotrophic waters piled up in the western Pacific, favouring the existence of a deep thermocline (An et al., 2018; Liu and Zhou, 2020). ENSO modelling indicates that this climate pattern could have been present in the past (Cane and Clement, 1999), and several studies recorded events probably attributable to ENSO variability in the Pleistocene (e.g. Pisias, 1978; Wang et al., 2000; Bordiga et al., 2014; Lupi et al., 2019).

In the tropical Pacific Ocean, SST reconstructions based on planktonic foraminifera oxygen isotopes and the Mg/Ca ratio, as well as alkenones (Liu and Herbert, 2004; de Garidel‐Thoron et al., 2005; Medina‐Elizalde and Lea, 2005; McClymont and Rossel‐Mele, 2005; Dyez and Ravelo, 2014) registered significant variations during the EMPT. The comparison of the eastern and western Pacific SSTs indicates a slow increase in the zonal temperature gradient during the Early Pleistocene, which became very strong at around 0.90 Ma (Dyez and Ravelo, 2014). A strong SST gradient intensifies the easterly trade winds and WC (Dyez and Ravelo, 2014; Kubota et al., 2021), indicating a shift from an El Niño‐ to a La Niña‐like mean state after the EMPT (de Garidel‐Thoron et al., 2005 Dyez and Ravelo, 2014).

During the Late Pleistocene, our planktonic foraminifera data indicate a weaker thermocline suggesting that the intensity of the NEC diminished. El Niño‐like conditions may have been responsible for weakening the NEC, enhancing a reduction of SSTs and less stable conditions in the western Pacific. In this respect, several changes in western Pacific SST were recorded during the latest Pleistocene (Ujiié, 2003; Jian et al., 2000; Bordiga et al., 2014) with phases of low SSTs (Gallagher, 2015). For instance, Bordiga et al. (2014) linked changes in calcareous nannofossil productivity and low SSTs during MIS 7–5 to ENSO perturbations (El Niño), resulting in a weak KC and the existence of the Kuroshio Current Extension in a ‘contracted state’ configuration. Oceanographic studies indicate that the intensity of the KC is related to the latitude of the NEC bifurcation (Weiss et al., 2021). During El Niño phases, the NEC bifurcation shifts northward, resulting in a weaker KC (Wu et al., 2019). The opposite situation occurs during La Niña events when the bifurcation moves southwards. In the western Pacific, the intensity of the NEC increases when the bifurcation point shifts southward and vice versa (Liu and Zhou, 2020). Thus, during El Niño events, both the NEC and KC are weak. If this is the case, in the later part of interval 2 (0.22–0.07 Ma), phases of weak KC during MIS 7–5 correspond to periods of less intense NEC.

### Assessing the effect of serpentinite mud volcanism on the planktonic foraminifera assemblage

The Fantangisña seamount is situated between 2018.22 and 3496.21 mbsl (Fryer et al., 2018b). Therefore, we maintain that the seamount is too deep to directly influence the ecological requirements and distribution of the planktonic foraminiferal assemblages at the investigated site.

Diversity data are very close for the serpentinite mud and the pelagic deposits. Diversity is an important parameter, as it indicates possible environmental stresses, with low species diversity indicating unfavourable environmental conditions, to which only a few opportunistic species are able to adapt (Gray, 1989). Similar and high values in diversity recorded for the two deposits suggest that the serpentinite mud volcanism did not affect the planktonic foraminifera fauna composition since a large number of species was recorded during both the active and quiescent phases of the seamount.

*Globigerinoides conglobatus* is a warm‐water mixed layer taxon (Kennett and Srinivasan, 1983; Hemleben et al., 1989). In our study, its pattern in abundance does not match those observed for the other mixed layer taxa. Specifically, this species is common in the pelagic layer (interval 2) and rare in the serpentinite mud deposits (interval 1). Contrary to the main species belonging to the *Globigerinoides* group, *G*. *conglobatus* possesses a calcite crust, making this species extremely resistant to dissolution (Hemleben and Auras, 1984). Furthermore, Hemleben et al. (1989) suggest that this species can inhabit greater depths despite being considered a mixed layer dweller. If this is the case, *G*. c*onglobatus* does not prefer strong water column stratification to migrate deeper. This could explain its lower abundance during periods of a well‐stratified water column at the studied site. However, we suggest that the high abundance of *G*. *conglobatus* within the pelagic layers is more likely due to increased dissolution during deposition as indicated by observed overgrowth, specimens’ fragmentation, and pore widening found on other planktonic species (see Appendix 1, Plate 4). Common *G*. *conglobatus* was also observed within the calcareous microfossil‐rich uppermost deposits overlying the summit of the near South Chamorro seamount (ODP Site 1200) as well as in a few preserved pelagic intervals within the serpentinite deposits found at the same site, and interpreted as a result of increased dissolution (Salisbury et al., 2002). In addition, the high abundance of *G*. *conglobatus* related to the dissolution of the planktonic foraminifera was also documented in the western Pacific (Berger et al., 1982; Ujiié and Ujiié, 2000).

Dissolution does not completely alter the original assemblage. This can be suggested by the presence of *G*. *rubescens* within the assemblage characterising the pelagic deposits. This species is highly susceptible to dissolution and is usually only preserved if dissolution is not severe (Berger et al., 1982). The pelagic sediments were deposited during a quiescent period of Fantangisña seamount in the Pleistocene (less than 0.61 Ma), during which dissolution of planktonic foraminifera resulted from deposition at a greater depth than 3000 mbsl. Conversely, when the seamount was active, serpentinite mudflows possibly allowed for the rapid burial of planktonic foraminifera, thus preventing dissolution of their tests.

### Benthic foraminifera

Most benthic foraminifera were recovered within the pelagic cover and the forearc sediments, situated above and below the serpentinite mud deposits. Here, benthic fauna are dominated by epifaunal forms, indicative of oligotrophic and well‐oxygenated bottom‐water conditions (Rodriguez et al.,^2018^). The assemblages show high abundances of *Nuttallides umbonifer*, common *Globocassidulina subglobosa* and tube‐shaped agglutinated species. *Nuttallides umbonifer* is a dissolution‐resistant species (Bremer and Lohmann, 1982) that thrives in oligotrophic and low temperature/salinity oxygenated bottom waters (Loubere, 1991; Gooday, 1993; Mackensen et al., 1995; Widmark and Speijer, 1997) with low input of phytodetritus (Smart and Gooday, 1997). Low productivity and organic carbon fluxes are also reflected by low abundances of *Epistominella exigua* and *Alabaminella weddellensis*, which are considered valuable proxies for pulsed food supply (Gooday, 1988; Lambshead and Gooday, 1990; Gooday, 1993). Moreover, buliminids and bolivinids are extremely rare, while no uvigerinids were recorded within the assemblage. Those benthic groups favour environments with high food availability and low oxygenation (Sen Gupta and Machain‐Castillo, 1993; Thomas, 1998; Zhu, 2001; Jorissen et al., 2007).

*Globocassidulina subglobosa* prefers deep water with moderately strong bottom currents (Mackensen et al., 1995; Poli et al., 2012) and high oxygen levels (>2 mL/L O_2_; Kaiho, 1994). Although *G*. *subglobosa* was found to be related to the phytodetritus content in the northeastern Atlantic and Antarctica (Smart, 2008; Poli et al., 2012), we consider that its typical abundance at our site is due to the presence of well‐oxygenated bottom‐water as also suggested by the low abundances of species indicative of high food supply rates.

Agglutinated foraminifera are an important part of the benthic community, with mostly epifaunal morphotypes recorded. Among them, tube‐shaped species are very common, indicating low organic matter flux and well‐oxygenated bottom waters (Gooday, 1994; Kuhnt et al., 1996).

Palaeoenvironmental conditions obtained from the benthic assemblage within the pelagic deposits confirm the existence of low productive surface waters, as also suggested by planktonic foraminifera. Similar conditions were also present before the serpentinite mud production, as indicated by the benthic assemblage within the forearc deposit.

## Conclusion

Planktonic and benthic foraminifera assemblages were analysed in 47 samples from IODP Expedition 366, Site U1498A, situated on the southern flank of Fantangisña seamount in the northwestern Pacific Ocean. The investigated site is influenced by the NEC, a westward wind‐driven surface current flowing between 8°N and 17°N. Previous biostratigraphic analyses on this site indicated that most of our studied interval covers the Early to Late Pleistocene. The distribution of foraminifera indicates the following palaeoceanographic parameters and changes:

(1) Cluster analysis on Pleistocene planktonic foraminifera resulted in two sample groups based on the ratio of thermocline‐dwelling species (e.g. *Globorotalia* spp.) to mixed layer dwellers (e.g. *G*. *ruber*, *Trilobatus* spp., *G*. *glutinata*, *G*. *rubescens*). These groups suggest fluctuations in the DOT during the Pleistocene. Cluster 1 corresponds to the oxidised pelagic sediments dated to the Middle–Late Pleistocene (post‐EMPT) and contains abundant mixed layer taxa and a good number of thermocline specimens indicating a weaker thermocline. Cluster 2 represents serpentinite mudflow deposits which are Early–Middle Pleistocene in age (during the EMPT). Mixed layer taxa dominate the assemblage, whereas thermocline dwellers are very low in abundance, suggesting a deep thermocline.
(2) We further conclude that the mudflow activity of Fantangisña seamount did not affect the distribution of the planktonic foraminifer community as the submarine volcanic edifice is located at great depth. This is supported by high diversity values recorded for the serpentinite mud deposit and their similarity to the planktonic foraminifera diversity data obtained for the pelagic cover. Despite that, the serpentinite mudflow could protect the planktonic assemblage from dissolution, allowing a fast burial after deposition. Conversely, post‐depositional dissolution at great water depths (>3000 mbsl) was observed in the pelagic layers when the serpentinite mud volcano was not active, as indicated by the high abundance of the dissolution‐resistant species *G*. *conglobatus*. Nevertheless, the dissolution was not so severe as to completely alter the foraminifera assemblage as inferred by the presence of the dissolution‐susceptible species *G*. *rubescens*.
(3) Variations in the DOT point to intensification/weakening of the NEC, associated with El Niño–La Niña events during the Early to Late Pleistocene in the northwestern Pacific. During the EMPT, a deep and stable thermocline as well as warm conditions, are recorded, possibly related to the intensification of the WC and the establishment of a La Niña phase in the western Pacific. Our results agree with previous studies documenting high SSTs and more stable conditions in the northwestern Pacific during the EMPT. The strengthening of the WC, in turn, results in an intensified NEC. In contrast, a weaker thermocline was recorded in the Middle–Late Pleistocene (following the EMPT), possibly corresponding to periods of low SSTs, which were documented from past studies in the western Pacific after the EMPT. The shoaling of the thermocline may reflect the persistence of El Niño conditions in the northwestern Pacific, which could be responsible for the weakening of the NEC.
(4) Benthic foraminifera are present in very small quantities in the serpentinite mud deposits, while their diversity is high in the pelagic and forearc units. Thus, the distribution of benthic foraminifera is severely influenced by serpentinite mud volcanism (mudflows and gas exhalations).
(5) The high abundances of epifaunal taxa (e.g. *N*. *umbonifer*) and low numbers of infaunal species (e.g. bolivinids, buliminids) suggest nutrient‐poor and oxygenated bottom waters in the pelagic and forearc deposits.

## Supporting information

Supporting information.

Supporting information.

Supporting information.

Supporting information.

Supporting information.

Supporting information.

Supporting information.

Supporting information.

Supporting information.

Supporting information.

Supporting information.

## Data Availability

The data that support the findings of this study are available in the supplementary material of this article
