# Supplementary material for: Foraminifera assemblages from Fantangisña serpentinite mud seamount in the NW Pacific Ocean during the Pleistocene (IODP Expedition 366)
Source: J Quat Sci. 2023 May 30;38(7):1103–27. doi: 10.1002/jqs.3532 (PMC10947277; doi:10.1002/jqs.3532)
Supplement: Supplementary file 11 — Supporting information. [file JQS-38-1103-s005.docx]

**Supporting Information**

**Figure S1.** Dendrogram of the cluster analysis using UPGMA method (Bray-Curtis similarity index) for planktonic foraminifera.

**Figure S2.** Principal Component Analysis (PCA) illustrating the distances between the clusters in a two-dimensional space formed by two axes corresponding to component 1 and component 2, which define the largest distance between datasets. Green lines show taxa responsible for the separation of the samples into clusters. 1a= subcluster 1a; 1b= subcluster 1b; 2= cluster 2. Taxa that contribute less to the definition of the clusters were removed from the plot for clearer visualization.

**Figure S3.** Non-metric Multidimensional Scaling (nMDS) using Bray‐Curtis similarity showing data into a 2D representation. 1a= subcluster 1a; 1b= subcluster 1b; 2= cluster 2.

**Table S1.** Dataset showing the total number of individuals identified for each planktonic foraminifera species, and the total number of reworked and indeterminate specimens. Grey areas= barren samples; orange areas= samples with a low number of planktonic foraminifera. Preservation for each sample was evaluated as follows: G= Good (mostly whole specimens, little evidence of overgrowth/dissolution, apertures often uncovered, well-preserved ornamentations, and/or no compression of the test; M= Moderate (most individuals identifiable at the species level, moderate overgrowth/dissolution, apertures often covered, ornamentations moderately preserved, and/or slightly compressed test; P= Poor (identification often difficult, substantial overgrowth/dissolution, ornamentations scarcely preserved, and /or severe test compression).

**Table S2.** Dataset showing the total number of individuals identified for each benthic foraminifera species, and the total number of reworked and indeterminate specimens. Grey areas= barren samples. Preservation for each sample was evaluated as follows: G= Good (mostly whole specimens, little evidence of overgrowth/dissolution, apertures often uncovered, well-preserved ornamentations, and/or no compression of the test; M= Moderate (most individuals identifiable at the species level, moderate overgrowth/dissolution, apertures often covered, ornamentations moderately preserved, and/or slightly compressed test; P= Poor (identification often difficult, substantial overgrowth/dissolution, ornamentations scarcely preserved, and/or severe test compression).

**Table S3.** Total number of planktonic foraminiferal individuals in the sediment and each analyzed size fraction.

**Table S4.** Total number of benthic foraminiferal individuals in the sediment.

**Table S5.** Datasets showing relative planktonic foraminifera species abundances, and results of the SIMPER analysis using the Bray-Curtis similarity. Additionally, minimum, maximum, average, and standard deviation values for all taxa in each cluster are shown, as well as the number of mixed layer and thermocline dwellers in each sample.

**Table S6.** Planktonic foraminifera diversity indices are shown for each sample.

**Table S7.** Dataset showing relative benthic foraminifera species abundances, the number of epifaunal-infaunal species in each sample. Additionally, a table showing the microhabitat preference of the identified benthic taxa.

**Appendices**

**Appendix 1.** Images of the most relevant planktonic foraminifera species (Plates 1-4).

**Appendix 2.** Images of the most relevant benthic foraminifera species (Plates 5-7).
